# Heterogeneity in the inter-tumor transcriptome of high risk prostate cancer

**DOI:** 10.1186/s13059-014-0426-y

**Published:** 2014-08-26

**Authors:** Alexander W Wyatt, Fan Mo, Kendric Wang, Brian McConeghy, Sonal Brahmbhatt, Lina Jong, Devon M Mitchell, Rebecca L Johnston, Anne Haegert, Estelle Li, Janet Liew, Jake Yeung, Raunak Shrestha, Anna V Lapuk, Andrew McPherson, Robert Shukin, Robert H Bell, Shawn Anderson, Jennifer Bishop, Antonio Hurtado-Coll, Hong Xiao, Arul M Chinnaiyan, Rohit Mehra, Dong Lin, Yuzhuo Wang, Ladan Fazli, Martin E Gleave, Stanislav V Volik, Colin C Collins

**Affiliations:** Vancouver Prostate Centre & Department of Urologic Sciences, University of British Columbia, 2660 Oak Street, Vancouver, BC V6H 3Z6 Canada; Bioinformatics Training Program, University of British Columbia, Vancouver, BC Canada; Michigan Center for Translational Pathology, Ann Arbor, Michigan USA; Department of Experimental Therapeutics, BC Cancer Agency, Vancouver, BC Canada

## Abstract

**Background:**

Genomic analyses of hundreds of prostate tumors have defined a diverse landscape of mutations and genome rearrangements, but the transcriptomic effect of this complexity is less well understood, particularly at the individual tumor level. We selected a cohort of 25 high-risk prostate tumors, representing the lethal phenotype, and applied deep RNA-sequencing and matched whole genome sequencing, followed by detailed molecular characterization.

**Results:**

Ten tumors were exposed to neo-adjuvant hormone therapy and expressed marked evidence of therapy response in all except one extreme case, which demonstrated early resistance via apparent neuroendocrine transdifferentiation. We observe high inter-tumor heterogeneity, including unique sets of outlier transcripts in each tumor. Interestingly, outlier expression converged on druggable cellular pathways associated with cell cycle progression, translational control or immune regulation, suggesting distinct contemporary pathway affinity and a mechanism of tumor stratification. We characterize hundreds of novel fusion transcripts, including a high frequency of ETS fusions associated with complex genome rearrangements and the disruption of tumor suppressors. Remarkably, several tumors express unique but potentially-oncogenic non-ETS fusions, which may contribute to the phenotype of individual tumors, and have significance for disease progression. Finally, one ETS-negative tumor has a striking tandem duplication genotype which appears to be highly aggressive and present at low recurrence in ETS-negative prostate cancer, suggestive of a novel molecular subtype.

**Conclusions:**

The multitude of rare genomic and transcriptomic events detected in a high-risk tumor cohort offer novel opportunities for personalized oncology and their convergence on key pathways and functions has broad implications for precision medicine.

**Electronic supplementary material:**

The online version of this article (doi:10.1186/s13059-014-0426-y) contains supplementary material, which is available to authorized users.

## Background

In recent years the application of next-generation sequencing to hundreds of prostate tumors has defined novel molecular subtypes and characterized extensive genomic aberration underlying disease initiation and progression [1,2]. Rearrangements of *ETS* transcription factors define approximately 50% of tumors [3], while mutations in the E3 ubiquitin ligase adapter *SPOP* and/or disruption to *CHD1*, a chromatin remodeling factor, have been reported in approximately 20% of tumors [4–6]. Both *ETS* rearrangements and *SPOP* mutations appear to be early events in prostate cancer development and proceed to influence the nature of future aberration, resulting in subtype-specific patterns of downstream genome rearrangement [6]. However, the glut of genomic and epigenomic aberrations accrued during progression continue to converge on characteristic ‘prostate cancer pathways’ with scant regard to molecular subtype: ultimately leading to a highly heterogeneous transcriptomic landscape, centered on an overactive androgen receptor (AR) signaling axis. This heterogeneity confounds attempts to advance beyond clinically-based nomograms for patient prognostication and to accurately stratify tumors for precision medicine. Furthermore, since driver mechanisms within individual prostate tumors are highly diverse (as evidenced by lack of highly recurrent mutations), specific events are in many tumors likely to be unique.

High-risk clinically localized prostate cancer, a potentially lethal disease, is diagnosed in up to one-quarter of patients [7], and tends to be highly rearranged at the genomic level, harboring multiple drivers [8]. Since previous sequence-based studies have focused predominantly on the genome or exome, we hypothesized that detailed transcriptome dissection of high-risk prostate tumors will reveal unique and contemporary driver aberration. In this study we characterized the transcriptomes of 25 high-risk primary prostate tumors including 10 neo-adjuvant treated tumors, identifying novel gene expression signatures and hundreds of fusion transcripts, some of which may be therapeutically tractable. Although highly heterogeneous, aberration converged on distinct cancer pathways and functions, with impact for future efforts to stratify patients for precision medicine. Finally we identified a previously unrecognized tandem duplicator genotype in *ETS*-rearrangement negative prostate cancer.

## Results

### Evidence of therapy response and resistance in neo-adjuvant treated tumors

**Figure 1:**
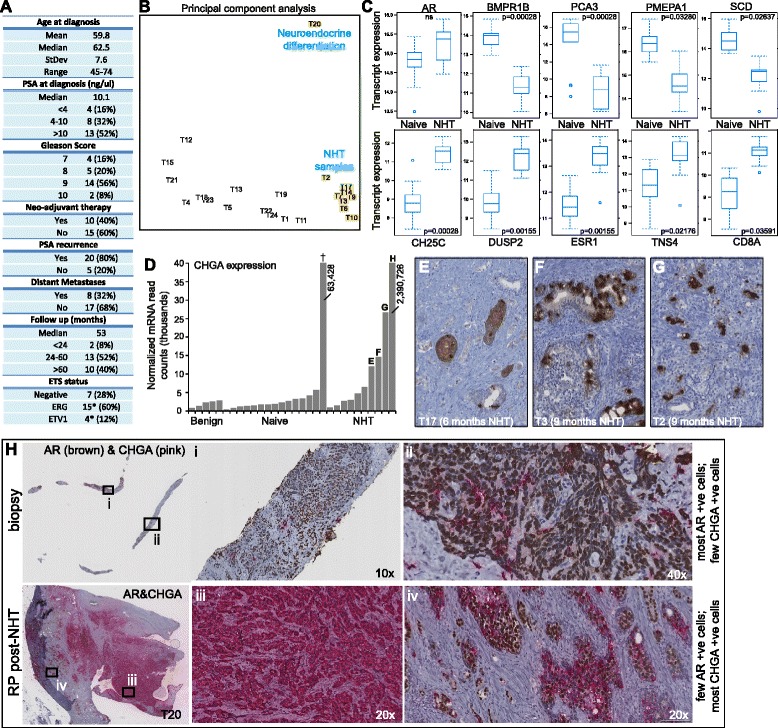
**Therapy response and resistance in neo-adjuvant hormone treated tumors. (A)** Breakdown of the patient cohort. **(B)** Principal component analysis using RNA-seq derived transcript expression (after removal of three outliers; see Additional file 2: Figure S1) demonstrating the global split between hormone-naive tumors and tumors treated with neo-adjuvant hormone therapy (NHT). **(C)** Representative genes that were significantly differentially expressed between hormone-naive and NHT tumors (DESeq comparison; Benjamini-Hochberg corrected *P* values). **(D)** Expression levels of the neuroendocrine prostate cancer marker *CHGA* in the cohort, showing elevated levels in NHT tumors. † indicates the unique hybrid adenocarcinoma-neuroendocrine tumor described in-depth in a separate study (ref 45). **(E**-**G)** CHGA protein staining showing diffuse positivity (<10% of cells overall) in selected NHT tumors with elevated CHGA mRNA expression levels. **(H)** Dual AR (brown) and CHGA (pink) staining in tumor T20 (with the highest mRNA expression of *CHGA* in **(D)**) showing evidence of neuroendocrine transdifferentiation, potentially in response to 8 months of NHT. The top panel shows the diagnostic biopsy, prior to treatment, showing few (pink) CHGA positive cells but predominant (brown) AR staining, while the bottom panel shows tumor T20 (at radical prostatectomy (RP)), demonstrating small cell morphology, widespread (pink) CHGA positivity and scant remaining AR positive foci.

We performed deep transcriptome sequencing (median 711× coverage), shallow whole genome sequencing (median 23× coverage), and aCGH copy number profiling on 25 high-risk primary prostate tumors, five matched adjacent-benign prostate tissues, and a patient-derived xenograft (PDX) originating from a needle biopsy of high-risk primary disease (Figure 1A; Additional file 1: Tables S1-3). Detailed follow-up information for more than 2 years was present for all but two patients: the majority of patients (20/25) had a PSA recurrence or never reached PSA nadir, and eight of these have so far progressed with distant metastases, emphasizing the selected high-risk nature of the cohort.

Ten tumors were exposed to neo-adjuvant hormone therapy (NHT) for 1 to 9 months, and global transcript profiling demonstrated a marked split between these samples and the untreated (hormone-naïve) tumors (Figure 1B; Additional file 1: Table S4; Additional file 2: Figure S1). Significantly downregulated genes in NHT tumours (DESeq comparison; Benjamini-Hochberg *P* <0.05) were typically androgen responsive (for example, *TMPRSS2*, *KLK3*, *BMPR1B*, *TPD52*) or steroidogenesis-related (for example, *DHCR24*, *SCD*), consistent with a reduction in androgen receptor activity (Figure 1C; Additional file 1: Table S5; Additional file 3: Text S1). This effect is in stark contrast to the frequent observation of AR reactivation in castration-resistant prostate cancer; which is often concomitant with the overexpression of *de novo* steroidogenic enzymes [9]. Upregulated genes in NHT tumors were linked to growth suppression and increased apoptosis (for example, *DUSP2*, *DUSP4*, *CCDC8*, *TNS4*) and also with cytotoxic T lymphocytes (for example, *CD8A*, *GZMK*), with the latter in line with previous reports demonstrating an increase in prostatic infiltration of lymphocytes post-hormone treatment [10,11]. However, although the data suggested that most tumors were responding to androgen deprivation therapy at the time of tumor collection, four tumors displayed elevated *CHGA* expression at the mRNA level (Figure 1D). CHGA is a marker of NEPC: an aggressive anaplastic subtype that is very rare at diagnosis but may emerge after long term androgen deprivation [12]. NEPC cells are AR-negative and are thought to arise via ‘transdifferentiation’ from AR-positive adenocarcinoma cells [13]. Elevated *CHGA* mRNA expression is of unclear significance in three of the four positive tumors since they exhibited <10% CHGA positive cells by immunohistochemistry (Figure 1E-G). However, one tumor (T20) exhibited widespread CHGA positivity, and morphology distinctive of neuroendocrine prostate cancer (NEPC) (Figure 1H). Dual immunohistochemistry for AR and CHGA on the same patient’s diagnostic biopsy (prior to NHT; diagnosis of adenocarcinoma, Gleason 4 + 5) revealed that biopsy contained few CHGA positive cells, while the prostatectomy sample (after 8 months of goserelin and flutamide) harbored few AR positive foci (Figure 1H, Additional file 2: Figure S2). Although we cannot exclude biopsy sampling bias, FISH on the prostatectomy sample confirmed the presence of the androgen-driven *TMPRSS2*-*ERG* rearrangement, suggesting an adenocarcinoma origin for the NEPC component within tumor T20 and the potential that this therapy resistant tumor was driven (at least in part) by NHT treatment (Figure 1; Additional file 2: Figure S2; Additional file 3: Text S1) [14–17].

### Outlier gene expression suggests contemporary tumor-dependent pathway affinity

**Figure 2:**
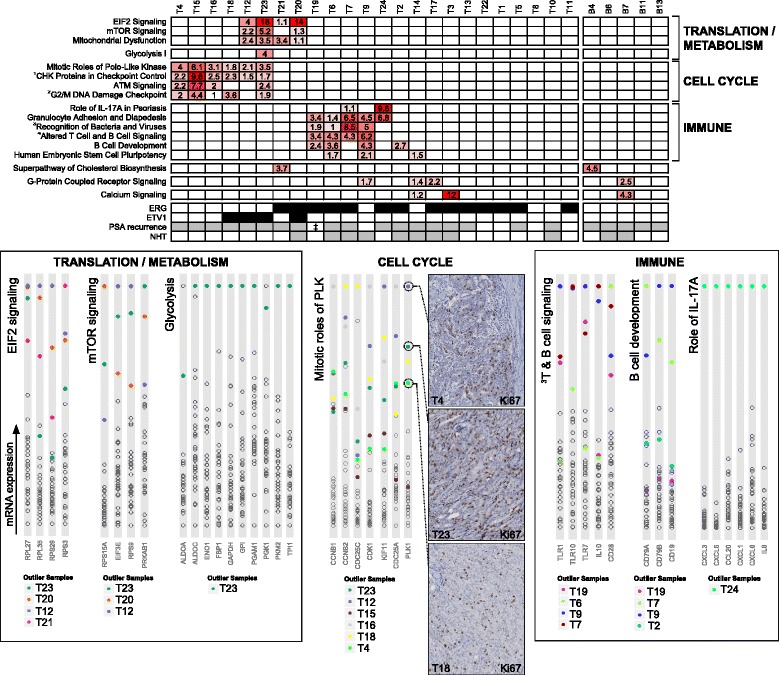
**Significant pathway enrichment of transcripts with outlier gene expression.** The heatmap in the top panel provides the pathway enrichment scores for representative canonical pathways in each tumor sample and benign tissue showing that outlier transcript sets from different tumors converge on distinct cellular functions (pathway score = -log_10_(Benjamini–Hochberg adjusted *P* value); only pathway scores >1 (that is, B-H <0.1) are shown). Note pathway names truncated from ^1^Role of CHK Proteins in Cell Cycle Checkpoint Control, ^2^Cell Cycle: G2/M DNA Damage Checkpoint Regulation, ^3^Role of Pattern Recognition Receptors in Recognition of Bacteria and Viruses, ^4^Altered T Cell and B Cell Signaling in Rheumatoid Arthritis. The bottom panel provides explorations of several key pathways, highlighting the expression distribution of representative genes within those pathways across the entire tumor cohort (full list of outlier gene sets within enriched pathways in Additional file 1: Table S8). Tumors with outlier gene enrichment within a given pathway are represented by colored circles. Immunohistochemistry images show high Ki67 indices in three tumors reflecting the probable high proliferation rate of tumors with outlier gene enrichment within cell cycle-related pathways (for additional images with larger fields see Additional file 2: Figure S3).

Despite elucidation of distinct molecular subtypes, the transcriptomic diversity of adenocarcinoma at prostatectomy is high, due in part to the protracted natural history of tumor development and the varying temporal relevance of a range of rare driver events. Concordantly, we did not observe broad gene expression differences between all tumor and benign samples at the significance levels used for the NHT comparisons described above (DESeq comparison; Benjamini-Hochberg *P* <0.05) (Additional file 1: Table S4). Nevertheless, the contemporary reliance (that is, at the time of sample collection) of individual tumors on particular cellular functions or signaling pathways for growth and survival should be overt at the transcriptome level. Therefore, to capture inter-tumor differences and similarities in pathway affinity, and to take advantage of the high dynamic range and absolute quantification inherent of RNA-seq data, we identified genes in each tumor that were expressed at an outlier level in that tumor and no more than one-third of the cohort (median outlier transcripts per sample = 437; Additional file 1: Table S6). This ‘recurrent’ outlier analysis allowed the detection of individual-specific outliers, and those that are more frequent. Remarkably, the outlier genes of 17/25 tumors showed significant enrichment (Benjamini-Hochberg corrected *P* <0.05) within at least one canonical pathway (Figure 2; Additional file 1: Tables S7 and S8).

Three main ‘clusters’ of pathway enrichment emerged (Figure 2). Pathways associated with cell cycle progression (for example, Mitotic Roles of Polo-Like Kinase) were significantly enriched in six samples, potentially indicating a subset of tumors with elevated proliferation rates: a claim further substantiated by high Ki67 indices in these tumors (Figure 2; Additional file 2: Figure S3). Enrichment was driven by the upregulation of several key regulators of the cell cycle, including *PLK1*, *CDC25A*, and *CDK1*, genes known to be associated with aggressive tumors, and with inhibitors in various stages of clinical development [18–20]. The translational control pathway ‘EIF2 Signaling’ was enriched in four tumors, with three-quarters also overexpressing genes involved in ‘mTOR Signaling’, a druggable pathway of considerable potential in prostate cancer [21]. This enrichment was caused in part by the upregulation of a range of ribosomal genes, a species frequently overexpressed in cancer [22]. Although increased ribosomal gene transcription may simply reflect a cell with higher proliferation, ribosomal biogenesis can itself be a driver of cell cycle progression, and several prominent oncogenes mediate their effects via the ribosome [22]. Interestingly, tumors with outlier gene enrichment in EIF2/mTOR also overexpressed genes related to mitochondrial dysfunction, and the tumor (T23) with the most significant enrichment in these three pathways had outlier expression of *AKT1* (notable since mitochondrial respiration defects can lead to the activation of AKT-mediated survival [23]; Additional file 2: Figure S3). Furthermore, T23 also demonstrated striking enrichment in the ‘Glycolysis I’ pathway, including marked upregulation of the Warburg-effect facilitator *PKM2* (evidence of coordinated regulation of this pathway was similarly evident in public data (Additional file 2: Figure S3)). Finally, a third group of tumors demonstrated enrichment of outlier gene expression within pathways associated with the immune system. Four of these tumors were exposed to NHT and distinct patches of infiltrating lymphocyte populations were evident by histology (Additional file 2: Figure S4). Accordingly, these samples had high expression of B cell markers (for example, *CD79*, *CD19*, *BLK*) and/or toll-like receptors (for example, *TLR1*), as well as T cell markers such as *CD4*, *CD8A*, and *CD3A*. Nevertheless, two tumors within the ‘immune group’ were untreated (T19 and T24) and although T19 had high expression of T and B cell markers, T24 had scant evidence of a significant population of invading lymphocytes either by histology or mRNA expression of immune cell markers (Additional file 2: Figure S4). High, unique expression of chemokines including *CXCL1*, *CXCL5*, and *IL8* in this tumor may therefore have tumor cell origins. Furthermore, it is worth noting that outlier genes from the five adjacent benign samples did not show enrichment in any of the three pathway clusters defined above, including benign samples matched to two tumors with high immune enrichment (Figure 2).

Since only two patients had died at time of writing, we evaluated instead the biochemical recurrence-free survival of different pathway groups. Despite the fact that the entire cohort was high-risk, the eight patients in the cell cycle and translation/metabolism groups fared significantly worse than all other patients (*P* = 0.0009; log rank test) (Additional file 2: Figure S5). Overall, these data promise a strategy to classify tumors based on contemporary druggable pathway affinity, which if corroborated in larger RNA sequence cohorts in the future, has potential to compliment patient stratification for precision medicine and provide insight into the functional consequences of heterogeneity.

### High frequency of ETS fusions, complex rearrangements, and disruption to tumor suppressors

DNA rearrangement is a hallmark of prostate cancer, causing distinctive chromosomal copy number alterations and creating oncogenic fusion genes [1]. Sentinel work over the past decade has defined the landscape of recurrent fusion genes in prostate cancer as limited to *ETS* gene rearrangements in approximately 50% of tumors, and rare (approximately 1%) rearrangement to *RAF* kinase family members [24]. However, non-recurrent events have attracted less attention, but may still have significant relevance within specific tumors. Indeed, genome breakpoints are likely to be an underestimated mutational mechanism in prostate cancer as they appear to be enriched within tumor suppressor genes, but analyses are complicated by the large number of passenger events (prostate tumors frequently harbor hundreds of genome rearrangements), and the false positives that are inevitable when predicting genomic events. Therefore, we focused our rearrangement analyses on our deep transcriptome data, since theoretically only genome breakpoints occurring within gene loci will be detectable and exon-exon junctions are less likely to cause mapping issues. We hypothesized that disrupted genes would tend to fall in prostate cancer-related pathways and that a fraction of non-recurrent fusion genes would have oncogenic potential.

**Figure 3:**
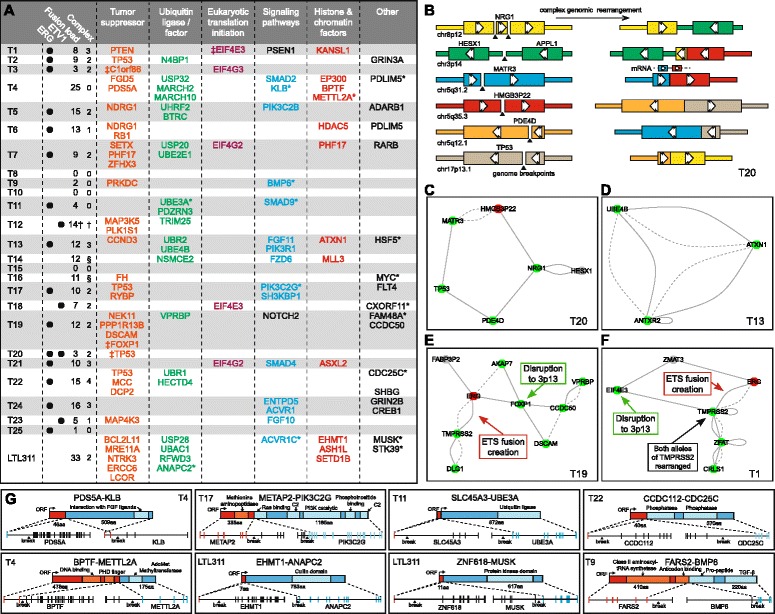
**The landscape of non-ETS fusion genes and complex genome rearrangements in high-risk prostate cancer. (A)** Depicts the number of expressed fusion genes (‘Fusion load’) and complex genome rearrangements (‘Complex’) detected in each tumor sample, and indicates selected genes involved in fusion events. Only genes with putative links to cancer are shown (full list of involved genes in Additional file 1: Table S9). Genes marked with * indicates a putative gain-of-function. † highly complex chromothripsis-driven rearrangements; ‡ involvement in complex rearrangement but not expressed; § DNA sequence coverage too low to elucidate complex rearrangements. **(B)** Schematic of the complex genome rearrangement (also known as chromoplexy [6]) in tumor T20 which lead to the disruption of TP53. Breakpoints in the six genes involved are indicated on the left, together with the final configuration on the right. This particular rearrangement led to the expression of just one fusion transcript (i.e. *MATR3*-*HMG3P22*). **(C**-**F)** Further schematics of complex genome rearrangements **(C)** shows the same rearrangement as **(B)**]. Green nodes indicate a gene is disrupted by rearrangement while red and gray indicate potential activating or neutral effects, respectively. Full edges represent a DNA rearrangement, and dotted lines indicate a rearrangement that was also detected in the RNA sequence data (that is, a fusion transcript was expressed). **(G)** Schematics of selected fusion genes with putative gain-of-function. Tumor ID is provided in each box, and major protein domains are annotated.

Using an integrated approach involving the fusion gene prediction tool deFuse [25], gene expression profiles and genome copy number data we identified 242 fusion transcripts arising from genome rearrangement in the 25 patient tumors and the PDX tumor (validation of 75 novel fusion transcripts was performed by PCR and Sanger sequencing; validation rate >95% [72/75]; Additional file 1: Table S9). Twenty-three out of 26 tumors expressed ≥1 fusion, at an average burden of 11 fusion transcripts. The majority (18/26; 69%) were positive for an *ETS* gene rearrangement (15 ERG, 4 ETV1; Figure 3A; Additional file 2: Figure S6); and *ERG* fusion junction read counts correlated with *ERG* gene expression (r = 0.82). Given the recent characterization of high frequency complex rearrangements (also termed ‘chromoplexy’) in prostate tumors [6], we searched for evidence that fusion genes were involved in complex rearrangements including >2 genome breakpoints. Despite shallower DNA sequence data, 210/242 fusions were evaluable (expressed in tumors with paired DNA sequence coverage >5×) for involvement in complex rearrangements, and we could identify DNA breakpoints for 169 (80%; Additional file 1: Table S9). There were 37 separate complex rearrangements involving 83/210 fusion transcripts (validation rate 100% [13/13]; Figure 3A-F; Additional file 1: Table S10), with 16 tumors containing ≥1 complex rearrangement. Of 17 evaluable *ETS* fusions, 14 were present in a complex rearrangement, and *ETS* rearrangements were involved in 38% (14/37) of complex events. It is of note that the NHT tumor (T20) with a significant NEPC component was positive for both *ERG* and *ETV1* rearrangements (by RNA sequencing and PCR validation); raising the possibility of multi-focal ‘collision’ cancers where only the *ERG* rearranged cells ‘transdifferentiated’ into NEPC (discussed further in Additional file 3: Text S1).

The majority of fusion genes were likely to result in loss-of-function of one or both partners through interruption and/or truncation of the coding sequence. In 18 tumors fusion gene events caused disruption to known or putative tumor suppressor genes, including genes associated with TP53 apoptosis (for example, *TP53* in four tumors; *PPP1R13B*), MAPK p38 apoptosis (for example, *MAP3K5*), cell cycle progression (for example, *RB1*, *CCND3*), DNA damage response (for example, *MRE11A*), and DNA architecture (for example, *PDS5A*) (Figure 3A; Additional file 1: Table S9). Indeed, using Ingenuity Pathway Analysis, the top canonical pathway enriched by the 344 unique fusion partners (that mapped to a protein coding gene) was the ‘Molecular Mechanisms of Cancer’ (Benjamini-Hochberg corrected *P* = 3.4 × 10^-5^) and the top disease/bio function was ‘Cancer: Solid Tumor’ (Benjamini-Hochberg corrected *P* = 1.66 × 10^-10^). Other classes of rearranged genes included E3 ubiquitin ligases and members of eukaryotic translation initiation complexes (Figure 3A). Particularly notable within the latter was disruption to *EIF4E3*, a gene residing within a genome rearrangement hotspot on chromosome 3p13 harboring three context-specific tumor suppressors *FOXP1*, *RYBP*, *SHQ1* [8,26]. *EIF4E3* falls in between *FOXP1* and *RYBP*, and is itself a purported tumor suppressor [27]. Although 3p13 is deleted in approximately 20% of prostate tumors, there is an enrichment of 3p13 deletions in *ERG* positive tumors [26]. Our data suggest this association may be partly underpinned by complex genome rearrangement, since two tumors appeared to have disruption to 3p14 simultaneously to *ERG* rearrangement (complex rearrangements in tumor T1 (*EIF4E3*) and tumor T19 (*FOXP1*); Figure 3E and F).

### Expression of non-ETS in-frame fusion genes

We searched for evidence of non-ETS fusions which could be oncogenic. Approximately 60 fusion genes were predicted to have an open reading frame across the fusion junction, while 45 were associated with outlier expression of the 3’ gene (Additional file 1: Table S9). Several candidates had theoretical gain-of-function (Figure 3G; Additional file 1: Table S9; Additional file 2: Figure S6), including genes within intracellular signaling cascades (for example, WNT or PI3K pathways). For example, a *PDS5A*-*KLB* fusion (tumor T4) led to 10-fold overexpression of a truncated, but in-frame, *KLB* transcript. KLB (beta-Klotho) is a tissue restricted single-pass transmembrane protein that acts a co-receptor for FGF family members and has been implicated in prostate cancer [28,29]. Expression of *KLB* in T4 is driven by *PDS5A*, a known tumor suppressor involved in DNA repair and sister chromatid cohesion, disruption of which may also confer a selective benefit. *METAP2*-*PIK3C2G* in tumor T17 resulted in the seven-fold upregulation of in-frame *PIK3C2G*, while *FARS2*-*BMP6* in tumor T9 may also be relevant since BMP6 is linked to invasion of prostate cancer cells [30]. The PDX LTL311 expressed a *ZNF618*-*MUSK* fusion transcript resulting in the expression of a transcript coding for the protein kinase domain of MUSK, a muscle-specific receptor tyrosine kinase that is not normally expressed in prostate.

Genes involved in cell cycle regulation were also potentially activated, including in hormone-naïve tumor T22 where a fusion was associated with six-fold overexpression of tyrosine phosphatase *CDC25C*. In LTL311 the ubiquitously expressed *EHMT1* likely drove overexpression of *ANAPC2*. ANAPC2 is a component of the anaphase promoting complex/cyclosome (APC/C), a cell cycle-regulated E3 ubiquitin ligase that controls progression through mitosis and the G1 phase of the cell cycle. Interestingly, the partners of *ANAPC2*, *CDC20* and *CDH1*, were also upregulated in this tumor (Additional file 2: Figure S6), and APC/C has a recently-documented important role in cancer, and can be inhibited with a small molecule (Tosyl-L-Arginine Methyl Ester). Another oncogenic ubiquitin ligase, *UBE3A* (aka E6-AP; marks TP53 for proteolysis degradation) was placed under control of the AR via fusion to the first exon of androgen responsive gene SLC45A3 in T11, although *UBE3A* was not expressed at an outlier level in this tumor at the time of prostatectomy. Tumor T11 had an intact *TP53* gene, but examination of copy number and mutation data (Additional file 2: Figure S6 and Additional file 3: Text S1) revealed that *TP53* was disrupted in over half of the high-risk cohort (Additional file 2: Figure S7).

### A recurrent tandem duplication genotype in prostate cancer

**Figure 4:**
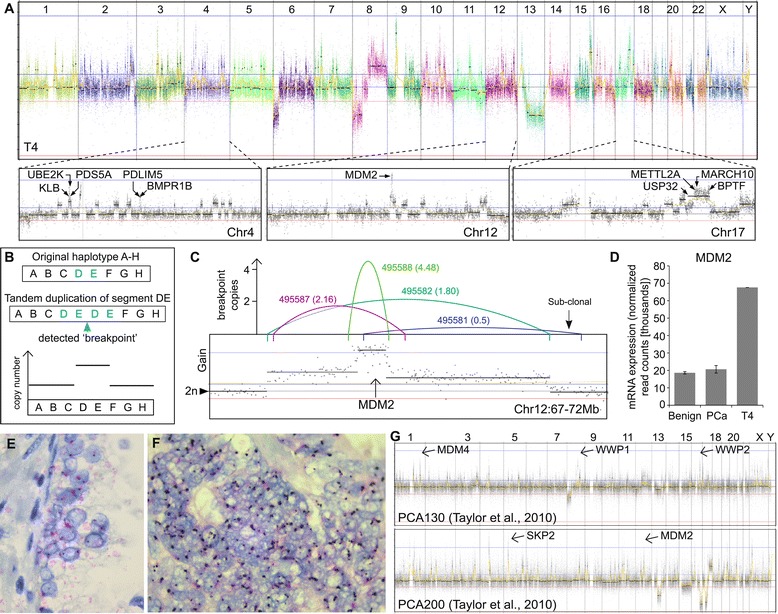
**A tandem duplication genotype in high-risk prostate cancer. (A)** Copy number profile of hormone-naïve tumor T4, showing focal gains across the entire genome, distinctive of tandem duplications. Key events discussed in the text are annotated. **(B)** Schematic illustrating how individual tandem duplications present at the genomic level. **(C)** Serial tandem duplications across the *MDM2* loci in T4. The copy number plot shows a focal high gain, with the colored lines representing segments that have been duplicated. Tandem duplication ID is indicated next to each colored line and in brackets is the estimated number of copies of each tandem duplication. For example, the genetic breakpoint of the most focal tandem duplication (green line) was detectable at a high frequency indicating multiple copies, suggesting that the breakpoint itself had been subsequently duplicated, potentially by the broader tandem duplications (pink and blue lines). Note that one breakpoint (purple line) was predicted at <1 suggestive of sub-clonality and highlighting the potential for continual evolution. **(D)** Transcript expression of MDM2 across the high-risk cohort showing elevated expression in tumor T4 relative to the other tumors and benign samples. **(E, F)** Chromogenic in situ hybridization of *MDM2* in benign tissue **(E)** and tumor **(F)** from T4 confirming *MDM2* amplification. Note the ‘clumps’ of staining in the tumor cells suggesting *MDM2* amplifications are proximally located (consistent with tandem duplication). **(G)** Copy number profiles from two tumors in a public dataset, which appear to harbor the distinctive pattern of focal gains across the entire genome (see Additional file 2: Figure S9 for further examples). Oncogenes within focal amplifications are annotated.

The two tumors expressing the greatest number of fusion genes were ETS negative (tumor T4 and LTL311; also *SPOP* wild type), consistent with Baca *et al.*’s work demonstrating a higher frequency of genome rearrangement in this tumor subtype compared to ETS positive tumors [6]. Remarkably, in the hormone-naïve tumor T4 all 25 expressed fusion genes were intra-chromosomal, and were predicted to have arisen through individual tandem duplication events. The copy number profile of T4 was uncharacteristic of prostate cancer, replete with hundreds of focal gains (Figure 4A). Overlapping focal copy gains with genome rearrangement predictions revealed an additional 216 tandem duplications spread across the entire genome of T4 (241 in total; validation rate 100% (13/13)) (Figure 4B; Additional file 1: Table S11). Several tandem duplication events had potential to contribute to cancer in T4, most notably the non-ETS fusion gene *PDS5A*-*KLB* (described above), and a high focal genome amplification of the *MDM2* gene loci (Figure 4C). The latter was caused by serial tandem duplication events across a roughly 3 Mb region of chromosome 12 (confirmed by CISH in Figure 4E and F). MDM2 is an oncogenic ubiquitin ligase whose action leads to the degradation of TP53 (similar to UBE3A). *MDM2* was expressed very highly at the mRNA level in T4, and may counteract the intact *TP53* gene in this tumor (Figure 4D). This is particularly intriguing in the context of the *UBE3A* fusion gene described above, as it conceivably represents a distinct mechanism of TP53 control in prostate tumors and suggests therapeutic strategies which relieve ubiquitin-mediated TP53 repression may have efficacy [31,32].

The prostate cancer copy number ‘signature’ of 8p loss and 8q gain was still present in tumor T4, but overlaid with tandem duplications. Furthermore the genome breakpoints of several tandem duplications were detectable only at sub-clonal levels (Figure 4C; Additional file 2: Figure S8) and had reduced copy number peaks relative to other gains. Together with the (serially-generated) amplification peaks of *MDM2* and *NRP2*, these data suggest that the tandem duplication genotype arose over time rather than in a single catastrophic event (c.f. chromothripsis [33]), and that there may be a specific susceptibility to tandem duplication in T4.

Interestingly, the genotype of T4 appears highly similar to a recently reported ‘tandem duplicator’ genotype in ovarian and breast cancer [34,35], raising the possibility of a pan-cancer mechanism, and recurrence beyond this singleton case in prostate cancer. Therefore, we searched for evidence of similar cases in two external cohorts of copy number profiles from localized and metastatic prostate tumors [5,8]. Although categorical detection of tandem duplications is not possible from copy number data alone, we identified five tumor profiles with tens to hundreds of focal copy gains highly reminisce of tumor T4 (Figure 4G; Additional file 2: Figure S9). Furthermore, it is of note that the five prostate tumors putatively identified with the tandem duplication genotype were ETS rearrangement negative, *CHD1* wild-type, and where genotyping was carried out, *SPOP* wild-type (tumor T4 was also *CHD1* and *SPOP* wild-type). As such it is probable that the tandem duplicator genotype is recurrent at low frequency in prostate tumors and may in fact comprise a distinct molecular class.

## Discussion

We have characterized the transcriptomic landscape of 25 high-risk prostate tumors. Focus on non-recurrent aberration revealed considerable inter-tumor heterogeneity, and a diverse range of novel potentially-driving fusion genes and outlier transcript expression signatures, some of which may be therapeutically exploitable. Non-recurrent events still converged on distinct cancer-related functions and pathways, a finding assuming increased significance given the advent of precision medicine and a renewed motivation for accurate patient stratification.

We identified a tandem duplication genotype in the ETS-negative tumor T4 that appears to be recurrent at a low level in prostate, breast, and ovarian cancers. In T4, tandem duplications resulted in several potentially driving events via oncogene amplification (for example, *MDM2*, *NRP2*), gene fusion (for example, *PDS5A*-*KLB*), or tumor suppressor disruption (for example, *EP300*). Inhibitors for MDM2 (and to a lesser extent NRP2) are in pre-clinical development [36,37], but the presence of multiple tandem duplications at sub-clonal proportions in T4 indicates a propensity for evolution in response to targeted therapy. The suggestion that tandem duplications are arising over multiple cell cycles helps distinguish the genotype from chromothripsis and chromoplexy, which are thought to occur in a single event, and are typically associated at the copy number level with multiple deletions (rather than focal gains) [6,33]. Evidence of recurrence of the tandem duplication genotype at low frequency in other prostate tumors, as well as in recent studies of breast and ovarian tumors [34,35] suggests the possibility of a common cause, probably in defective DNA maintenance [38]. Interestingly, prostate tumors with evidence of the tandem duplication genotype were all ETS and *CHD1* wild type, hinting at a distinct and novel molecular subtype. This hypothesis requires rapid confirmation in additional tumors, since the evident aggression of the genotype demands an early detection strategy.

Several high-risk tumors expressed unique, but potentially functional, non-ETS fusions. Non-ETS fusion transcripts have been previously identified in prostate tumors [39–41], but have been understudied due to their non-recurrence, and the predicted loss-of-function of the vast majority. Their clinical relevance requires further elucidation, and much will be revealed through transcriptome sequencing of advanced prostate tumors underway as part of the SU2C Dream Team efforts. However, it is interesting to speculate that the lack of androgen responsive promoters for the majority of potentially functional non-ETS rearrangements identified here may confer a benefit when under the stress of androgen blockade. Although the lower depth of our DNA sequencing precluded precise clonality estimates, several of the potentially functional fusions had read counts suggestive of sub-clonality, and it will be interesting to monitor whether they will be preferentially selected or lost over the course of disease. It may ultimately become possible to therapeutically exploit unique fusions such as those identified here, but in the near future non-ETS fusions have potential to complement patient stratification for precision medicine (for example, fusion of a PI3K subunit could imply stronger rationale for pathway inhibition). In parallel to the patient sequence cohort, we identified several potentially functional fusion genes in the ETS-negative PDX LTL311. This xenograft tumor was derived from a needle biopsy specimen obtained at diagnosis [13], and given the protracted disease course of prostate cancer it is therefore conceivable that target discovery and *in vivo* testing of personalized therapies is possible very early in disease course, at least for the highest risk cases.

The inability to determine whether a given aberration has historical or contemporary significance is a major drawback of genome-centric studies. However, as appropriate patient selection rapidly becomes a necessity for clinical trial design, it is imperative that we understand contemporary dependence on oncogenic or druggable pathways. Studies of outlier gene expression can give insight into contemporary drivers, and has considerable precedence in prostate cancer (for example, discovery of ETS fusions and SPINK1 subtype [42]), but the high dynamic range and absolute quantification afforded by transcriptome sequencing offers new opportunities not available to previous microarray-based approaches. In our study, we revealed highly statistically significant enrichment of outlier gene expression within distinct cellular pathways associated with metabolism, translation, cell cycle, and the immune system. These associations may reflect differing pathway reliance of individual tumors and therefore a rationale for discrete therapeutic strategies (for example, mTOR inhibition), although further functional studies are clearly required. Interestingly, where comparisons to matched benign tissue were possible, outlier gene pathway enrichment appeared to be specific to the tumor foci, even the immune-related pathways where one might assume lymphocyte infiltration to be prostate-wide. Some outlier gene signatures, such as the coordinated upregulation of glycolysis enzymes or the expression of specific chemokines, were detected in just a single tumor, highlighting both the remarkable diversity of gene usage, and the requirement for further characterization of individual tumor transcriptomes.

The logistical challenge of obtaining longitudinal tumor samples in patients with prostate cancer has long hampered research into therapy resistance. Although the application of ‘liquid biopsies’ to late-stage patients will undoubtedly reveal novel mechanisms of resistance, the study of neo-adjuvant treated primary tumors has proven to be a partial solution, leading for example to the discovery of the adaptive stress-response as an effective drug target [43]. Our study revealed a marked therapy effect in the 10 NHT tumors, and evidence of early drug resistance in one tumor (T20) via probable transdifferentiation of adenocarcinoma to neuroendocrine prostate cancer (NEPC). We recently reported a patient-derived xenograft model of primary adenocarcinoma which upon androgen ablation rapidly ‘transdifferentiates’ to complete NEPC via an adaptive response [13]. It seems likely that this is the situation in tumor T20, and underscores the urgent need to develop better biomarkers to monitor for early resistance.

## Conclusions

Through the first deep transcriptome sequencing study of prostate tumors we have revealed surprising levels of inter-tumor heterogeneity converging on key functions and pathways, and conferring significant implications for precision medicine. Our study emphasizes the value of focusing on the individual rather than the cohort, especially when profiling extreme phenotypes, since we identified a diverse range of novel potentially-driving fusion genes, outlier transcript expression signatures and an aggressive tandem duplicator genotype.

## Methods

### Sample collection and sequencing

Prostate tissue was collected from high-risk patients undergoing radical prostatectomy and snap frozen according to the current Vancouver General Hospital pathology protocol. All patients signed a formal consent form approved by the ethics board, and in accordance with the Helsinki Declaration. High-risk cases were selected for this study by meeting any of the following criteria: Gleason ≥8, PSA ≥20, or clinical stage T3a and above. Hematoxylin and eosin (H&E) stained FFPE and frozen sections were reviewed by an expert pathologist (LF) to identify blocks with highest tumor content. For each frozen block used, a 5 μm slide was first taken for H&E staining, then 4 × 100 μm sections were taken for DNA and RNA isolation, before a second 5 μm slide was taken for H&E staining. Each H&E slide was required to have tumor content >50% in order for a tumor to proceed for sequencing. Additionally, we included the patient-derived xenograft LTL311 in our sequencing cohort. LTL311 is derived from a needle biopsy of high-risk primary adenocarcinoma [13]. This tumor was included in the study to evaluate the suitability of modelling high-risk disease from biopsy tissue prior to prostatectomy.

For DNA isolation, digestion of 100 μm snap-frozen tumour tissue with 0.2 mg/mL Proteinase K (Roche) in digestion buffer (50 mM NaCl, 10 mM Tris-HCl (pH 8.3), 1 mM EDTA and 0.5% SDS) was carried out overnight at 55°C. Samples were incubated with RNase solution at 37°C for 30 min and treated with protein precipitation solution followed by isopropanol precipitation of the DNA. The DNA was further purified by Phenol:Chloroform:Isoamyl Alcohol (25:24:1), and precipitated by adding 1/10th volume of 3 M sodium acetate and 2.5 volumes of 100% ethanol, before re-suspension in TE. RNA from snap-frozen tissue was isolated using the mirVana Isolation Kit from Ambion (AM 1560). DNA and RNA sequencing was performed on Illumina HiSeq 2000 at BCCA Michael Smith Genome Sciences Centre according to standard protocols. Four high-risk tumors sequenced on GAII in a previous study [44,45] were re-sequenced on HiSeq 2000 for this study.

### Array comparative genomic hybridization copy number profiling

A total of 0.5 μg of each genomic DNA was fluorescently labeled by following the NimbleGen enzymatic labeling protocol which employs Cy3 and Cy5 labeled random nanomers (TriLink Biotechnologies), a heat fragmentation step at 98°C for 10 min, and amplification with Klenow fragment 5′-3′exo- (New England Biolabs). Five micrograms of each Cy5-labeled sample was co-hybridized with 5 μg of Cy3-labeled human male reference DNA (Promega Corp) on Agilent SurePrint G3 Human Catalog CGH 8 × 60 K or 4 × 180 k slides following the Agilent Oligonucleotide Array-Based CGH for Genomic DNA Analysis Protocol v6.2. Arrays were scanned with the Agilent DNA Microarray Scanner, and quantified with Feature Extraction 10.5.1.1. CGH processed signal was uploaded into Biodiscovery Nexus CGH software v7, where quality was assessed and data were visualized and analyzed. Data is available at GEO accession number GSE55016.

### Sequence data mapping and processing

Raw sequence data are available at The European Nucleotide Archive (ENA), accession number PRJEB6530. DNA-seq reads were aligned onto the human reference genome (hg19/GRCh37) using BWA (0.5.9-r16) [46] allowing 1 nt mismatch at most in a 24 nt seed. For RNA-seq, reads were mapped onto the hg19 genome and exon-exon junctions by splice-aware aligner Tophat [47], using the known gene model annotation from Ensembl release 62. Reads with an unmapped mate or multi-mapped location were filtered out using Bamtools [48] and PCR or sequencing optical duplicates were marked and removed by Picard [49]. Using NCBI dbSNP build 132, multiple sequence local realignment around InDels and base quality recalibration was performed by GATK (The Genome Analysis Toolkit) [50] to correct likely misalignments. For DNA and RNA sequencing data of all specimens, SNVs/InDels were identified and filtered by GATK [51] to achieve high-confidence sites (strand bias, base quality, mapping quality, and position bias were taken into account). Additionally for RNA-seq data, we used samtools [52] to call SNVs/InDels, and retained as high-confidence only those sites which were concordant between both GATK and samtools results. All variants were annotated with genic regions and potential consequences on protein-coding sequences using the tool AnnoVar [53]. The effect of non-synonymous SNVs on protein function was assessed using Condel [54], a method which integrates several predictive tools (for example, SIFT, Polyphen2, MutationAssessor). To prioritize variants we first filtered against dbSNP build 137 (non-flagged only) and the five adjacent benign samples sequenced in this study, and then only considered variants detectable in both DNA and RNA reads (that is, expressed variants). Finally we excluded identical variants concurrently predicted in more than two samples as likely artefact. Mutated genes present in either Cosmic Cancer Gene Consensus [55] or reported to be a ‘Mut-Driver Gene’ [56] are shown in Additional file 2: Figure S10 and Additional file 1: Table S12. Mutation frequency was calculated based on DNA-seq reads alone. Reads of reference and variant were counted after local realignment and duplicate removal.

Based on the alignment of RNA-seq reads, gene expression profiles for each sample were calculated based on the gene annotation (Ensembl release 62). Only reads which were unique to one gene and exactly corresponded to gene structure were assigned to the corresponding genes. Raw read counts were normalized by R package DESeq [57], which was designed for gene expression analysis of RNA-seq data across all samples. Transcript expression profiles for all samples are provided in Additional file 1: Table S4. DESeq was also used to compare transcript expression between neo-adjuvant treated and hormone naïve tumors (after exclusion of the two tumors with NEPC components (tumors T16 and T20)), using a Benjamini-Hochberg corrected *P* value. To detect outlier gene expression across the cohort we used the Generalized Extreme Studentized Deviate (ESD) test [58] with an upper bound of 15 (half of the cohort size). Additionally, in order to filter out background noise and minimize artifact detection, we calculated the average background noise for each sample based on the coverage of inter-genic and intronic regions (which should be largely absent from pure RNA-sequence data). After subtracting background noise, we required that an upregulated outlier gene have a sequence depth greater than 10X, while for a downregulated outlier gene, the average sequence depth of non-outliers must also be greater than 10X. Pathway and functional enrichment analyses of outlier genes was performed using the Ingenuity Knowledge Base (Ingenuity Systems [59]).

### Identification of fusion genes and genome rearrangements

We used the deFuse algorithm [25] to predict rearrangements in RNA sequence libraries. Since there were approximately 8,000 unfiltered defuse predictions of chimeric RNAs after analyses of the 31 RNA sequence libraries (Additional file 4: Table S13), we filtered predictions according to the following criteria: a fusion gene candidate: (1) must be predicted to have arisen from genome rearrangement, rather than via a readthrough event; (2) must be predicted in no more than two sequence libraries (with the exception of ETS fusions; this step removes recurrent artifacts); (3) must map unambiguously on both sides of the predicted breakpoints (that is, no multi-mapping reads); (4) must not map entirely to repetitive elements; (5) must be detected in >5 reads (either split or spanning). This step reduced predicted fusion genes to <1,000, and candidates were then further prioritized by fulfilling any of the following secondary criteria: mapping to edge of copy number aberration, differential exon expression either side of breakpoints; outlier expression of 3′ gene in that sample relative to others. Although this stringent filtering strategy has potential to remove some true positives, we felt that preferable to an elevated false discovery rate. To identify complex genome rearrangements underlying predicted fusion RNAs we applied the nFuse pipeline [60] to RNA and DNA sequence libraries. Validation was performed by PCR across the predicted fusion junctions in cDNA or gDNA (oligonucleotide sequences provided in Additional file 1: Table S9 and S10). All amplification products were sequenced with an ABI PRISM 310 Genetic Analyzer to confirm identity.

### Immunohistochemistry

Immunohistochemical staining was conducted on serial sections (5 μm thick) by Ventana autostainer model Discover XT TM (Ventana Medical System) with enzyme labeled biotin streptavidin system and solvent resistant DAB Map kit by using 1/50 of CHGA rabbit monoclonal antibody # AC-0037 (Epitomics, Inc.), 1/200 concentration of AR (N-20) antibody # SC-816 (Santa Cruz Biotechnology), and 1/500 of anti-Ki-67 rabbit monoclonal antibody clone SP6 (Thermo Scientific™ Lab Vision). For dual staining a Blue Map kit was used with 1/500 of CHGA mouse monoclonal antibody cat# MAB5268 (Chemicon) and 1/50 concentration of AR (N-20) antibody cat # SC-816 (Santa Cruz Biotechnology). For negative controls the primary antibodies were replaced with the corresponding species normal immunoglobulin G. Previously tested tissue samples from our tumor bank were used as positive controls.

Chromogenic *in situ* hybridization (CISH) was carried out using the Ventana discovery ultra-automated slide stainer two color method. After standard pre-treatment of formalin-fixed, paraffin-embedded human prostate cancer specimen, the tissues were subjected to protease digestion for 8 min, followed by incubation with an *MDM2* DNP labelled DNA probe and a Chromosome 12 DIG labelled DNA probe for 6 h. Detection was carried out with Ventana’s ultraView SISH DNP Detection Kit and Red ISH DIG Detection Kit. Finally the slides were counterstained with Hematoxylin II and blueing reagent. Fluorescence *in situ* hybridization (FISH) for *ERG* gene rearrangement was carried out using a previously documented protocol [61] and scored manually by an expert pathologist (RM).

## Additional files

Additional file 1:
**Tables S1-S12.**
Additional file 2:
**Figures S1-S10.**
Additional file 3:
**Text S1.**
Additional file 4:
**Tables S13-S18.**
