# Supplementary material for: Heterogeneity in the inter-tumor transcriptome of high risk prostate cancer
Source: Genome Biol. 2014 Aug 26;15(8):426. doi: 10.1186/s13059-014-0426-y (PMC4169643; doi:10.1186/s13059-014-0426-y)

Figure S1: Global transcript profiling reveals outlier samples and a split between neo-adjuvant hormone treated and non-treated (hormone-naive) tumors. A) Principal component analyses (PCA) performed on all transcripts. PCA#1 shows three initial outliers, including the previously-published rare hybrid tumor T16 (Wu *et al.*, 2012), which were removed before performing the PCA in Figure 1B) The gene signature of neo-adjuvant hormone therapy response evaluated in the MSKCC dataset of 131 primary prostate tumors (Taylor *et al.,* 2010) demonstrating that up-regulation of selected ‘pro-apoptotic’ and ‘anti-growth’ genes appear to correlate with better disease-free survival. Images are screenshots from the cBioPortal for Cancer Genomics.

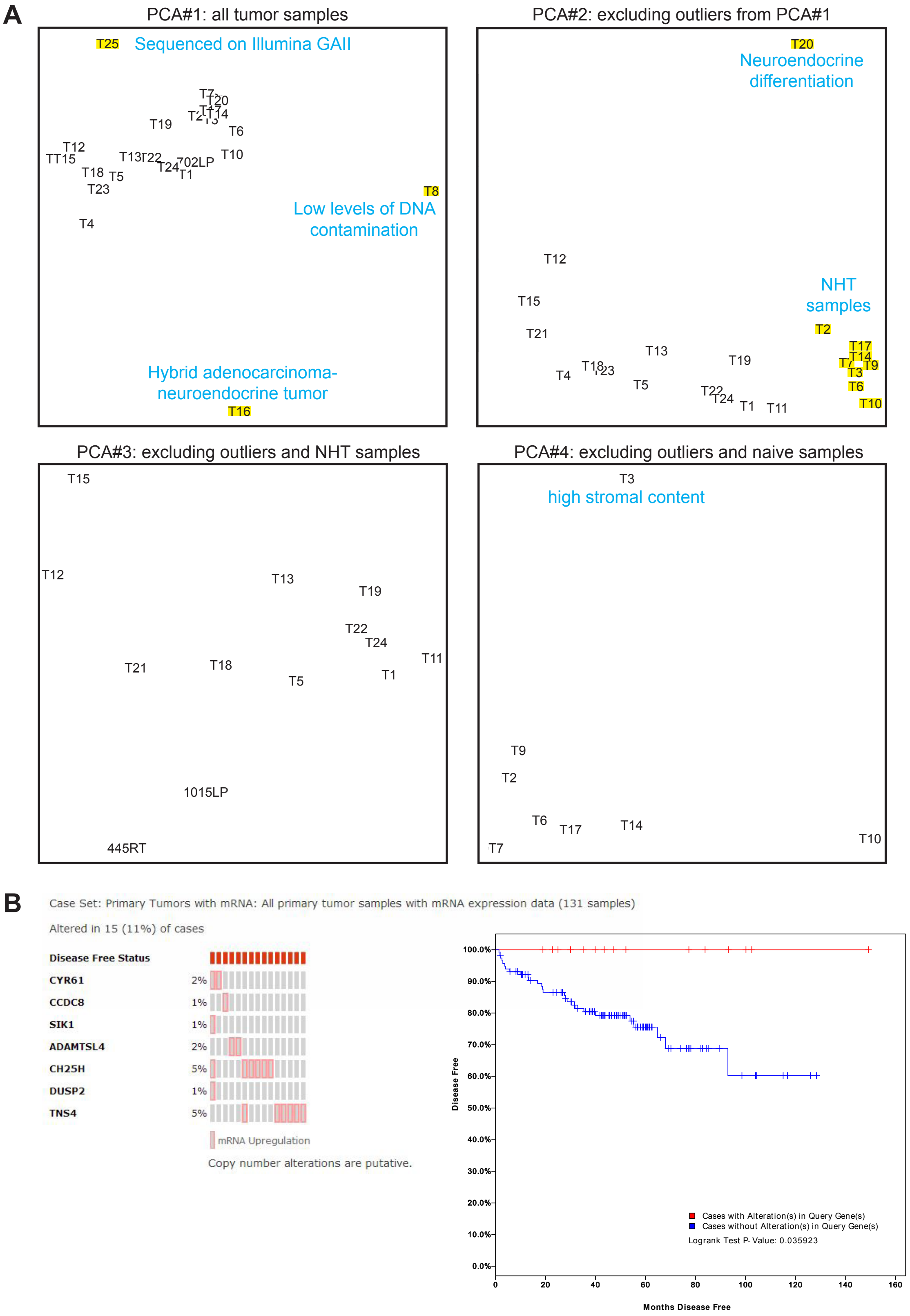

Figure S2: Additional immunohistochemistry images demonstrating evidence of neuroendocrine transdifferentiation in response to neo-adjuvant hormone therapy in tumor T20 (further to Figure 1H). A) Dual AR (brown) and CHGA (pink) staining in T20 highlighting a region with mixed positivity for AR and CHGA. B) H&E staining of T20. C) FISH staining of the ERG rearrangement in the NEPC regions of T20 showing positivity, suggestive of adenocarcinoma origins. Yellow staining shows co-localization of break-apart probes indicating wild-type ERG, while distinct red or green probes demonstrates an ERG rearrangement (Mehra *et al.*, 2008). D-E) H&E staining of the diagnostic biopsy from 8 months prior to collection of tumor T4; AR and CHGA IHC from this biopsy is shown in Figure 1H.

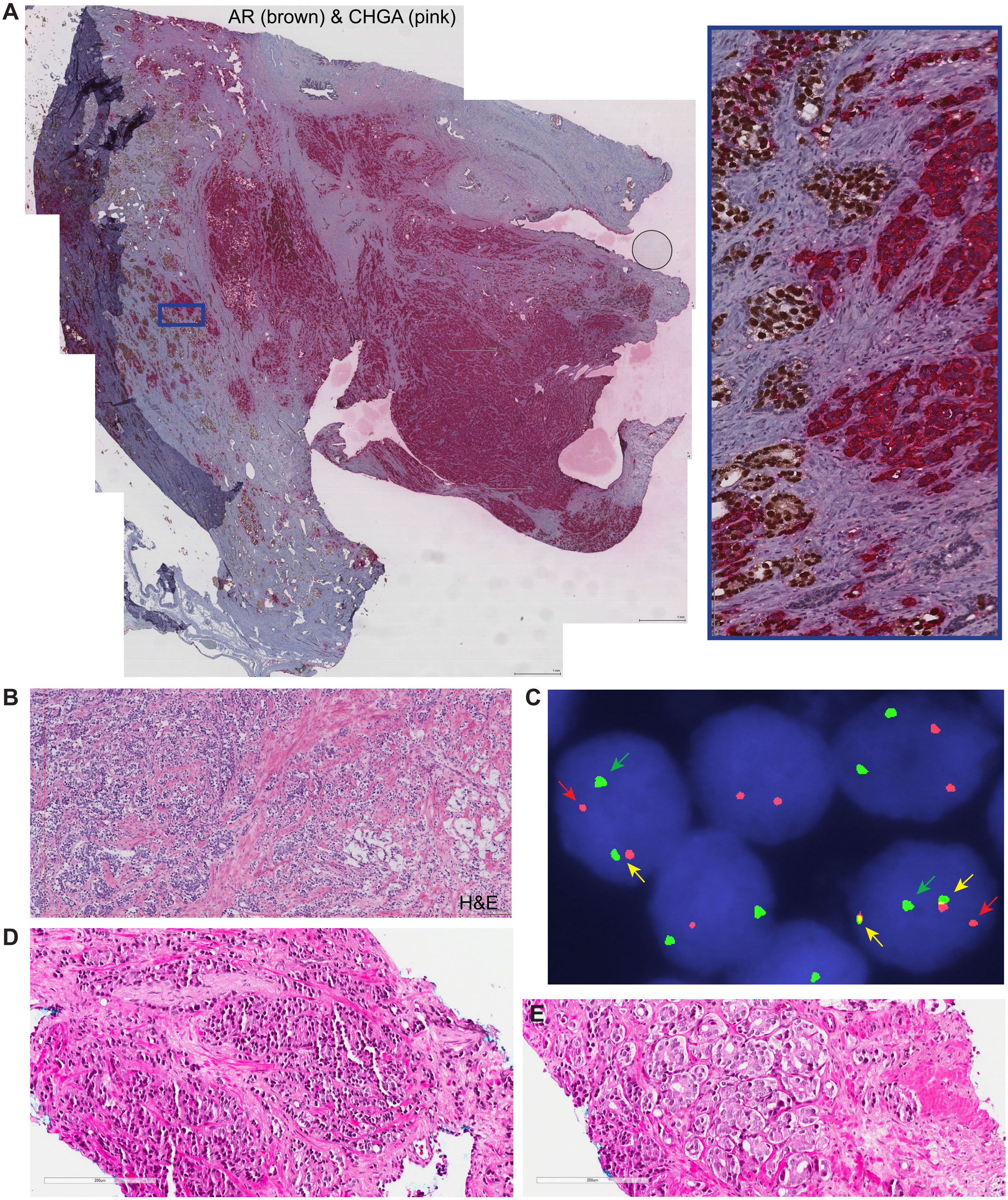

Figure S3: Support for pathway enrichment of outlier transcripts. A-C) High Ki67 staining in 3 tumors, further to the images provided in Figure 2. D) AKT1 and PKM2 expression is high in T23, the tumor with enrichment within the 'Glycolysis I' pathway. E) The outlier genes which lead to the enrichment Glycolysis I enrichment demonstrate evidence of co-expression in the MSKCC dataset of 216 prostate tumors, suggesting overall pathway co-ordination. Images are screenshots from the cBioPortal for Cancer Genomics and 'disease state' reflects metastatic (red) versus localized (blue) tumors; note tendency toward up-regulation in metastatic samples. F) Exploration of further sets of outlier genes (the sets shown in Figure 2) in the same MSKCC dataset, providing evidence of pathway co-regulation.

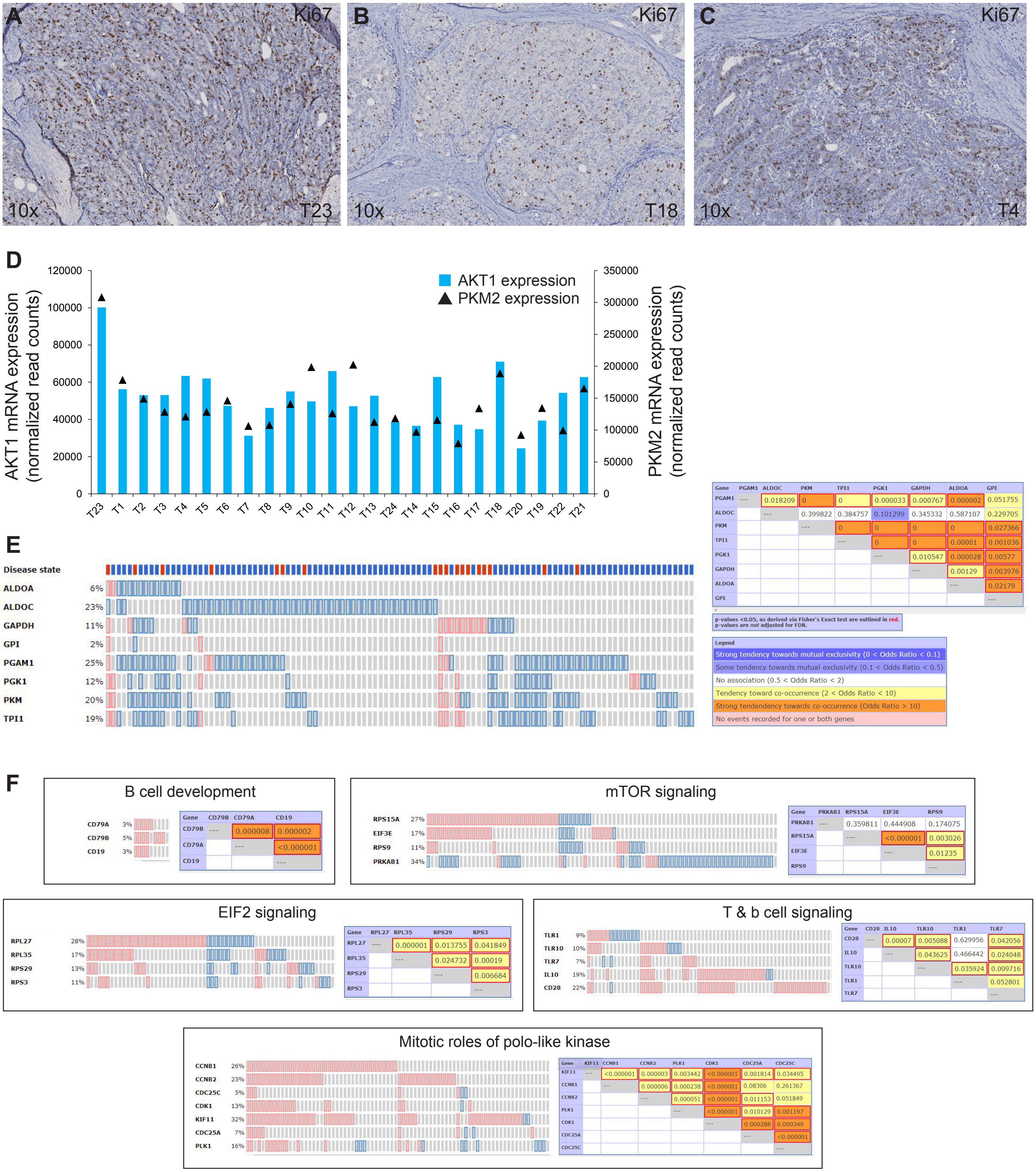

Figure S4: Support for pathway enrichment of outlier transcripts. A-B) Evidence of large lymphocyte patches within two of the samples with enrichment in immune-related pathways. C) Lack of histological evidence for immune infiltration in tumor T24. D) mRNA expression of typical immune cell markers across the cohort, showing high expression in tumors with immune pathway enrichment (red) consistent with immune cell infiltration, but low expression in T24 (blue), suggesting that tumor cells were expressing the chemokines outlined in Figure 2. E) High levels of stromal infiltration evident in H&E stains of T3 likely responsible for the significant ‘Calcium Signaling’ pathway enrichment. F) Expression of two representative genes that drove the ‘Calcium Signaling’ enrichment, highlighting their absence from other samples.

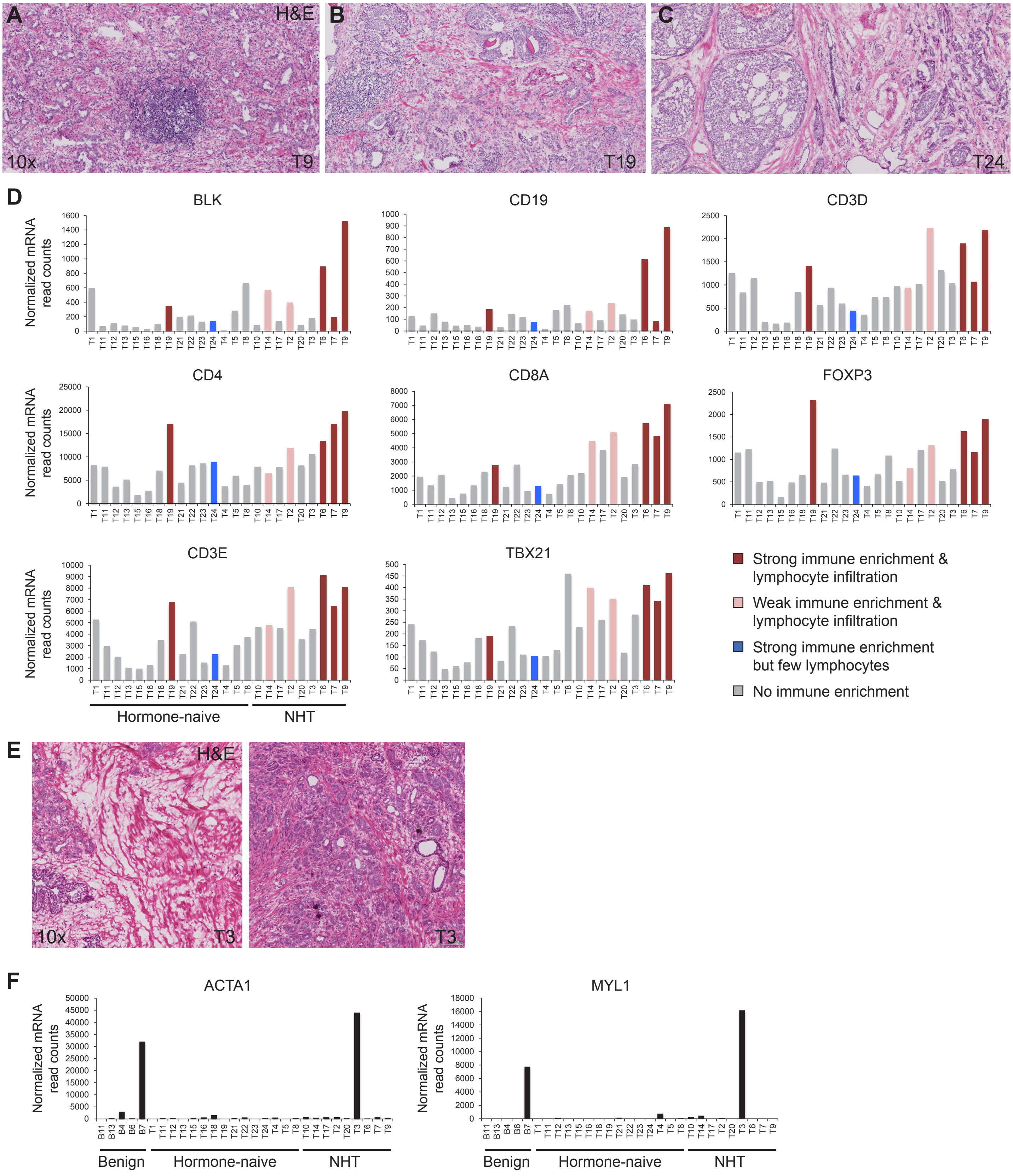

Figure S5: Kaplan-Meier plots showing differences in biochemical recurrence-free survival between tumors with differing pathway enrichment of outlier genes.

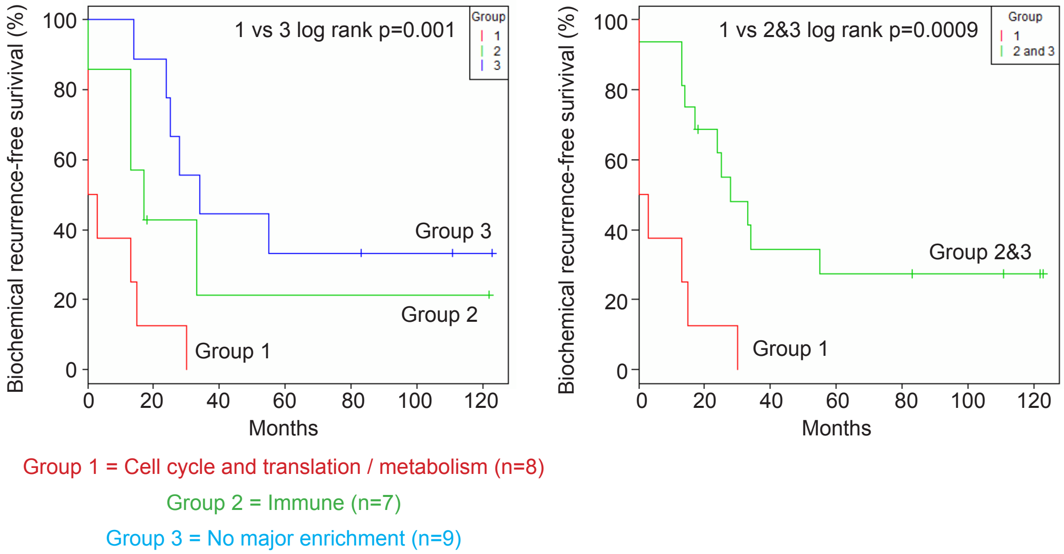

Figure S6: Fusion transcripts and complex genome rearrangement. A) ETS gene expression and ETS fusion junction read counts (transcriptome) across the cohort. B) Additional examples of complex genome rearrangement (chromoplexy). Green nodes indicate a gene is disrupted by rearrangement while red and grey indicate potential activating or neutral effects, respectively. Full edges represent a DNA rearrangement, and dotted lines indicate a rearrangement that was also detected in the RNA sequence data (i.e. expressed). C) Copy number status of significant prostate cancer genes across the cohort (aCGH data was unavailable for the 4 samples in grey). D) Additional selected fusion genes with potential gain-of-function (further to Figure 3G). Tumor ID is provided in each box, and major protein domains are annotated.

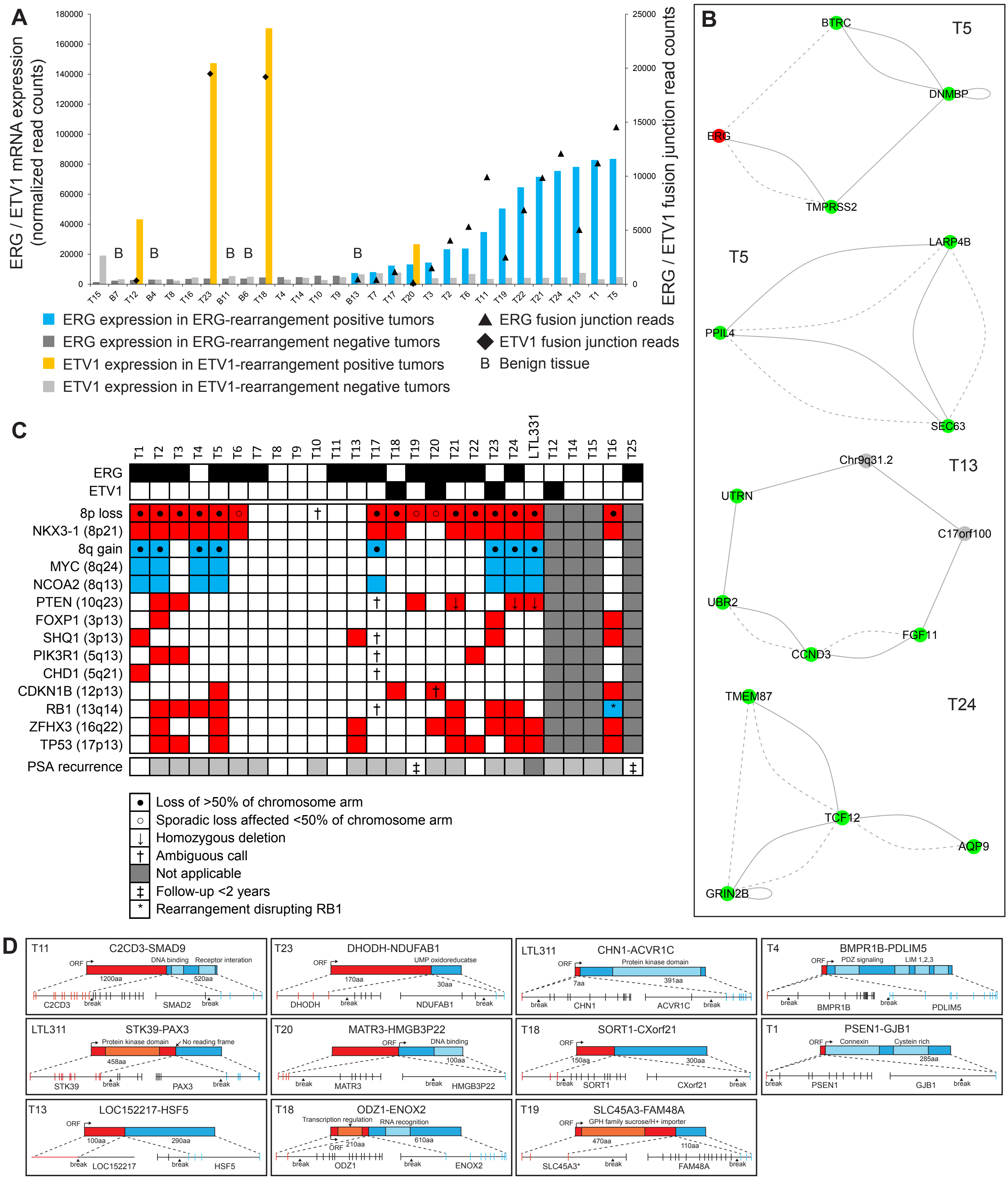

Figure S7: TP53 disruption through genome rearrangement, copy number loss and mutations. A) Plot of TP53 expression across the cohort with differing disruption to the gene annotated. B) Location of the three mutations in TP53 within a schematic of the TP53 protein. C) Copy number calls across chromosome 17 in tumors harboring a TP53 genomic deletion (red indicates a loss; blue a gain)

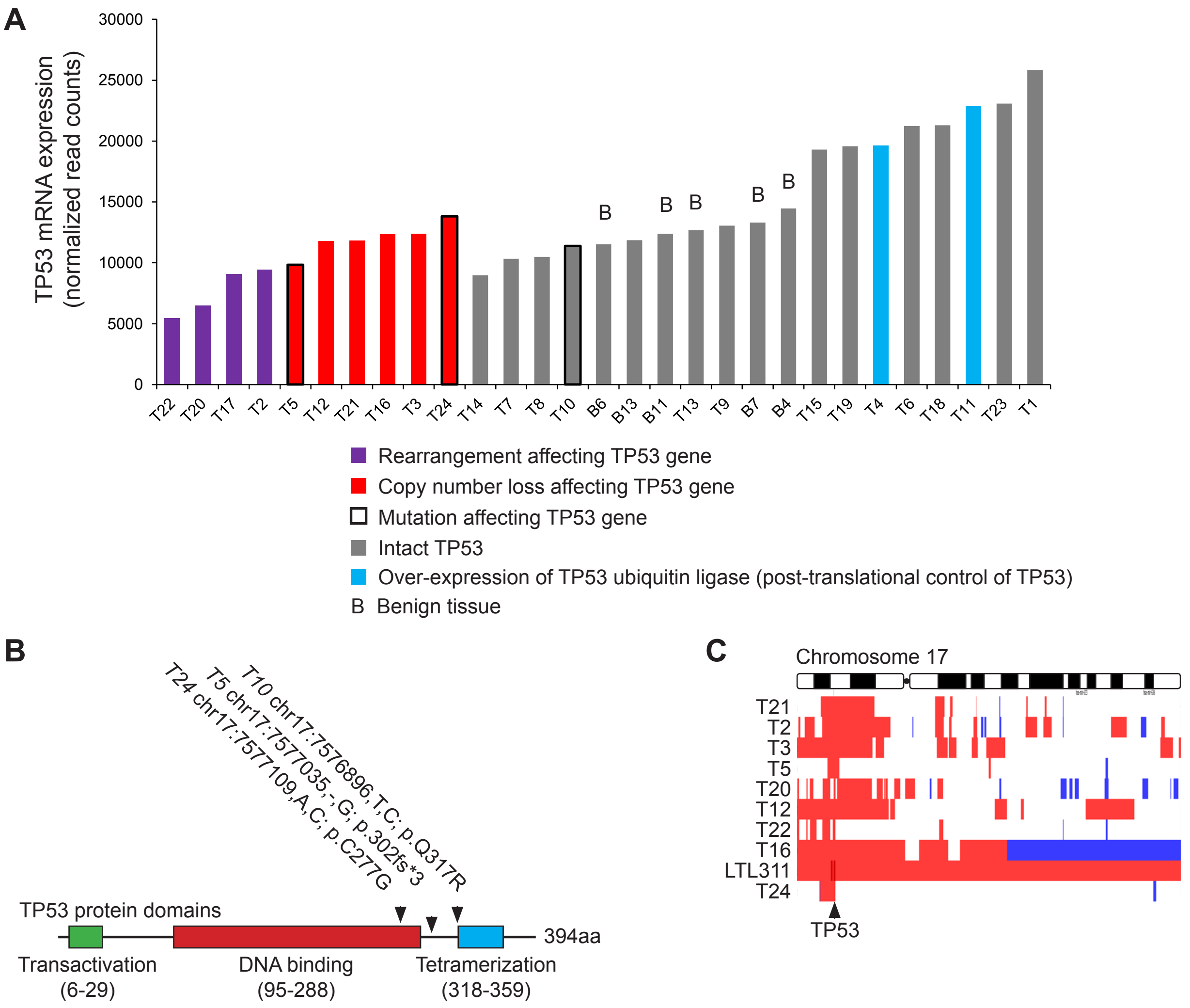

Figure S8: Tandem duplication genotype in tumor T4 A) Circos plot of the genomic rearrangements in T4. The inner-most track shows copy number changes (red=gain; green=loss), blue lines represent tandem duplications predicted from DNA sequence data, grey lines represent expressed fusion genes. B) An additional image of the MDM2 CISH result showing amplification in T4. C) Length of the tandemly duplicated segments in T4. D) Further examples (to Figure 4C) of genomic regions harboring multiple tandem duplications. The copy number plots show focal gains, with the colored lines representing segments that have been duplicated. Tandem duplication ID is indicated next to each colored line, and in brackets are the estimated number of copies of each tandem duplication.

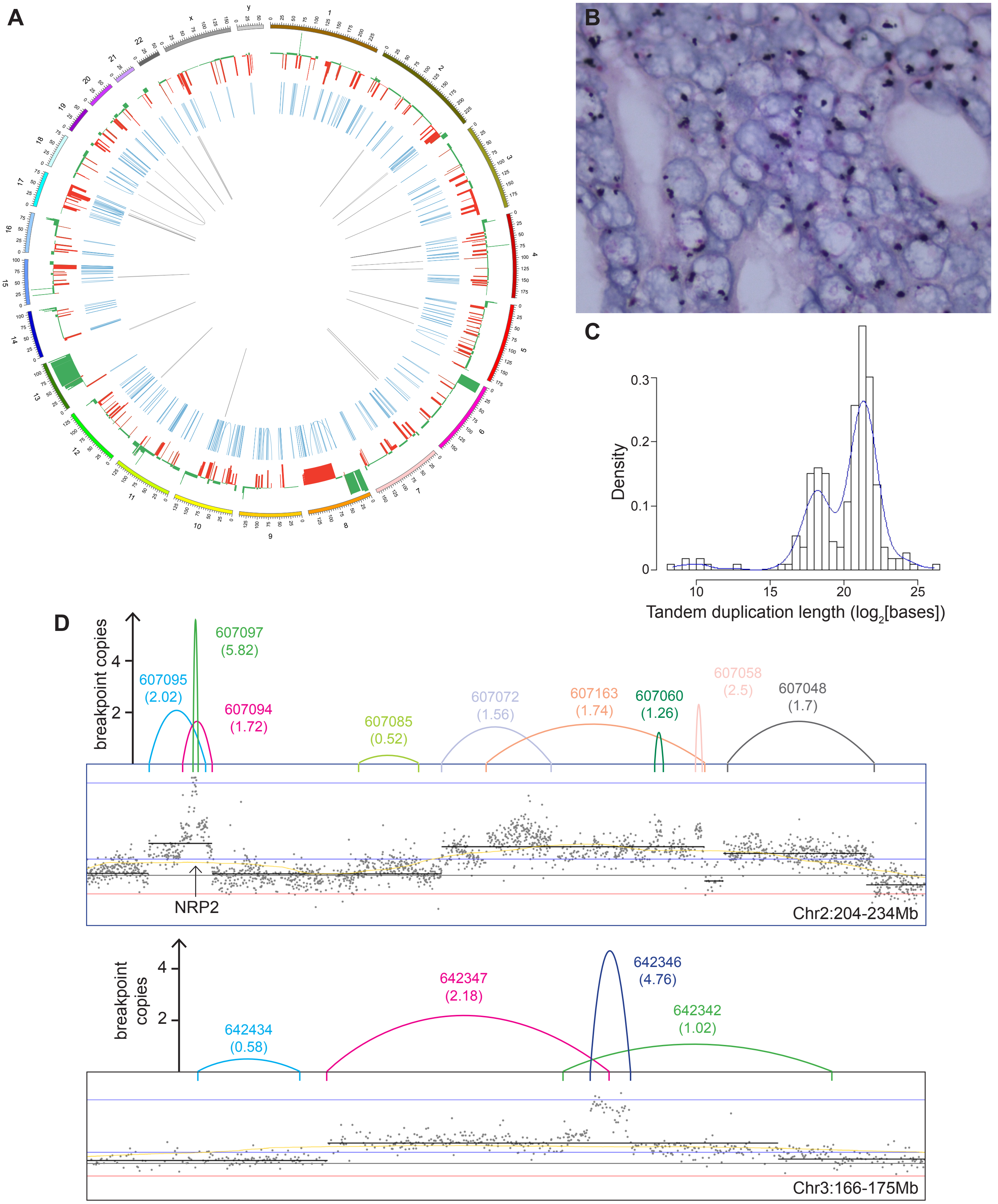

Figure S9: Recurrence of the distinctive tandem duplication genotype in other prostate tumors. The copy number profiles of five tumors (from two studies of localized and metastatic prostate cancer [Taylor *et al.,* 2010; Grasso *et al.*, 2012) which harbored tens to hundreds focal gains similar to the profile of tumor T4. Prostate cancer molecular subtype status is indicated on the right, although for the top two profiles it was inferred from copy number and expression data only. There were no mutations in TP53, PTEN or SPOP reported in the bottom 3 tumors (from the Grasso *et al.* 2012 study). +/- refers to a heterozygous deletion.

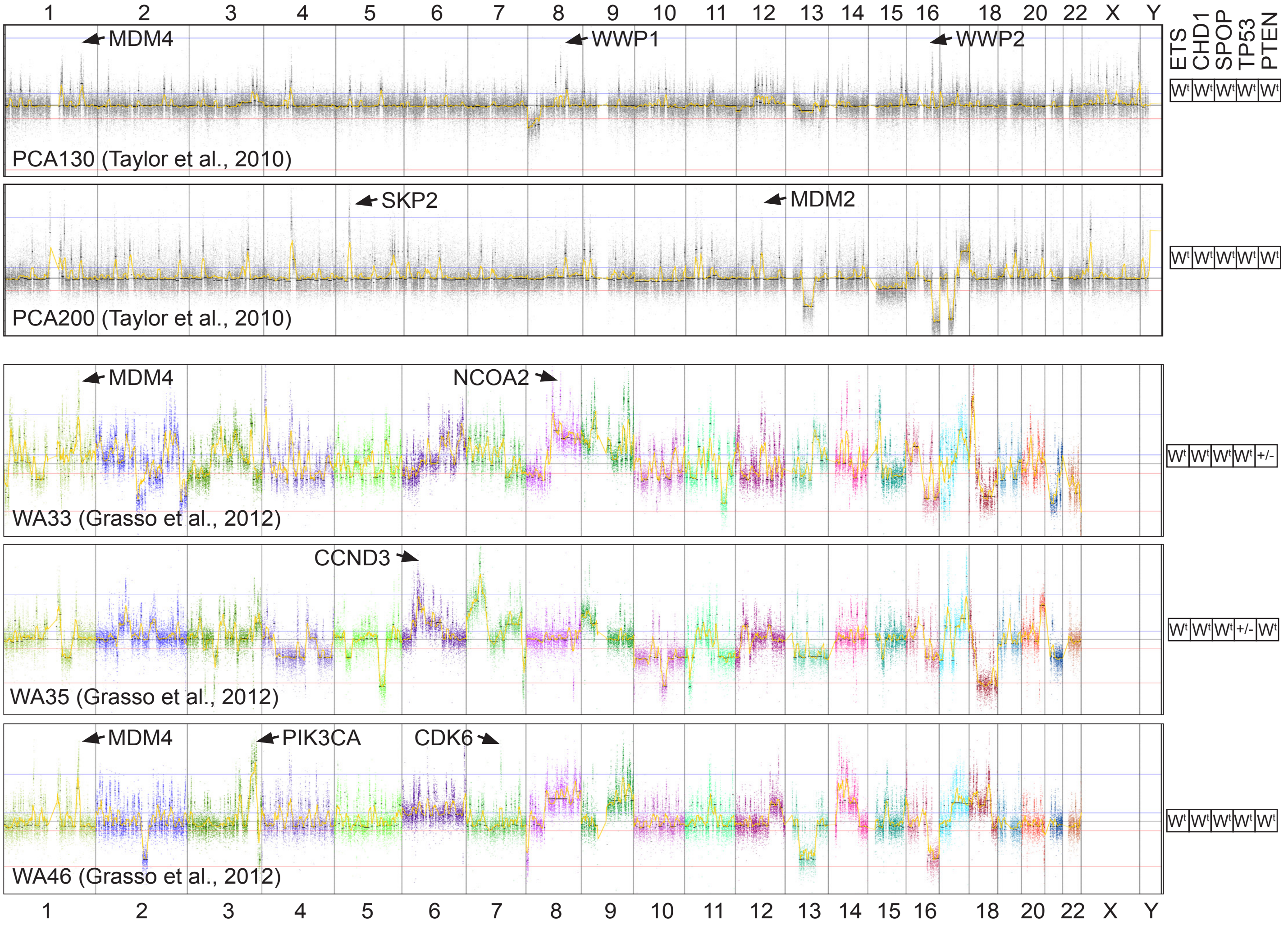

Figure S10: Expressed non-synonymous variants falling in cancer genes.

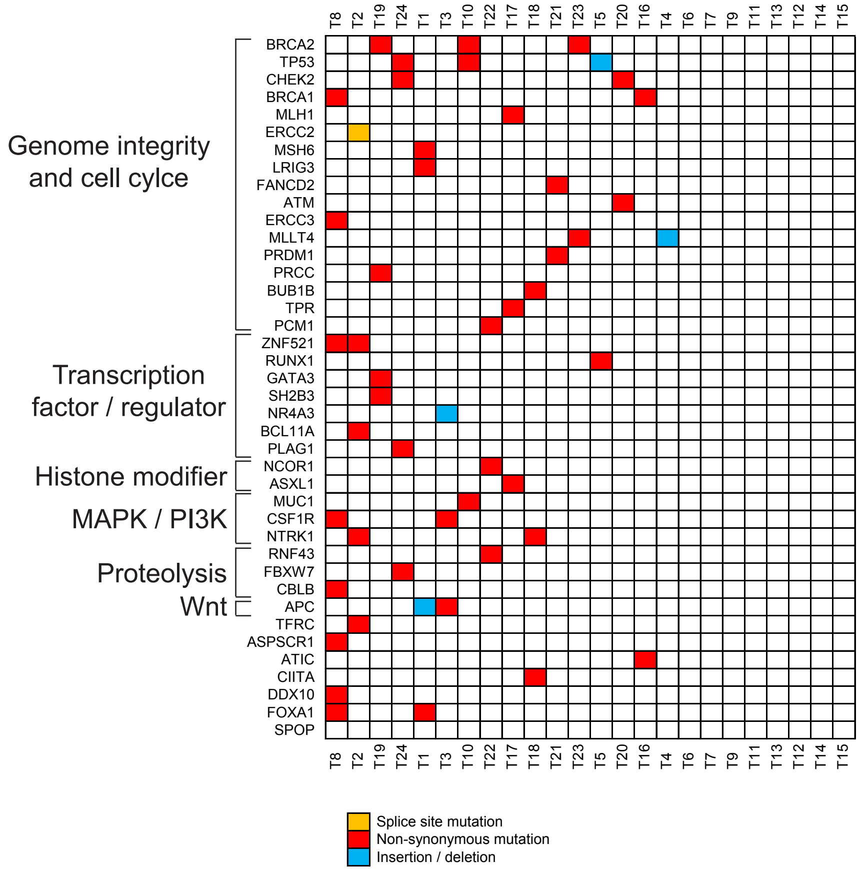

Figure S11: Integration of genomic variants with outlier gene expression. A) Expression of NCOA2 across the cohort, showing outlier expression in three samples with copy number gains at the NCOA2 loci. B) Example of correlation between a recurrent InDel within a miRNA binding site, and outlier expression of that gene (i.e. samples with potential disruption to the miRNA binding site exhibited higher expression of the transcript). C) Examples of correlation between recurrent genomic variants within promoter regions and outlier expression of that gene. Note that for B) and C) a cancer relationship is specifically not implied, since they are likely to represent germline events (although they are not present in dbSNP).

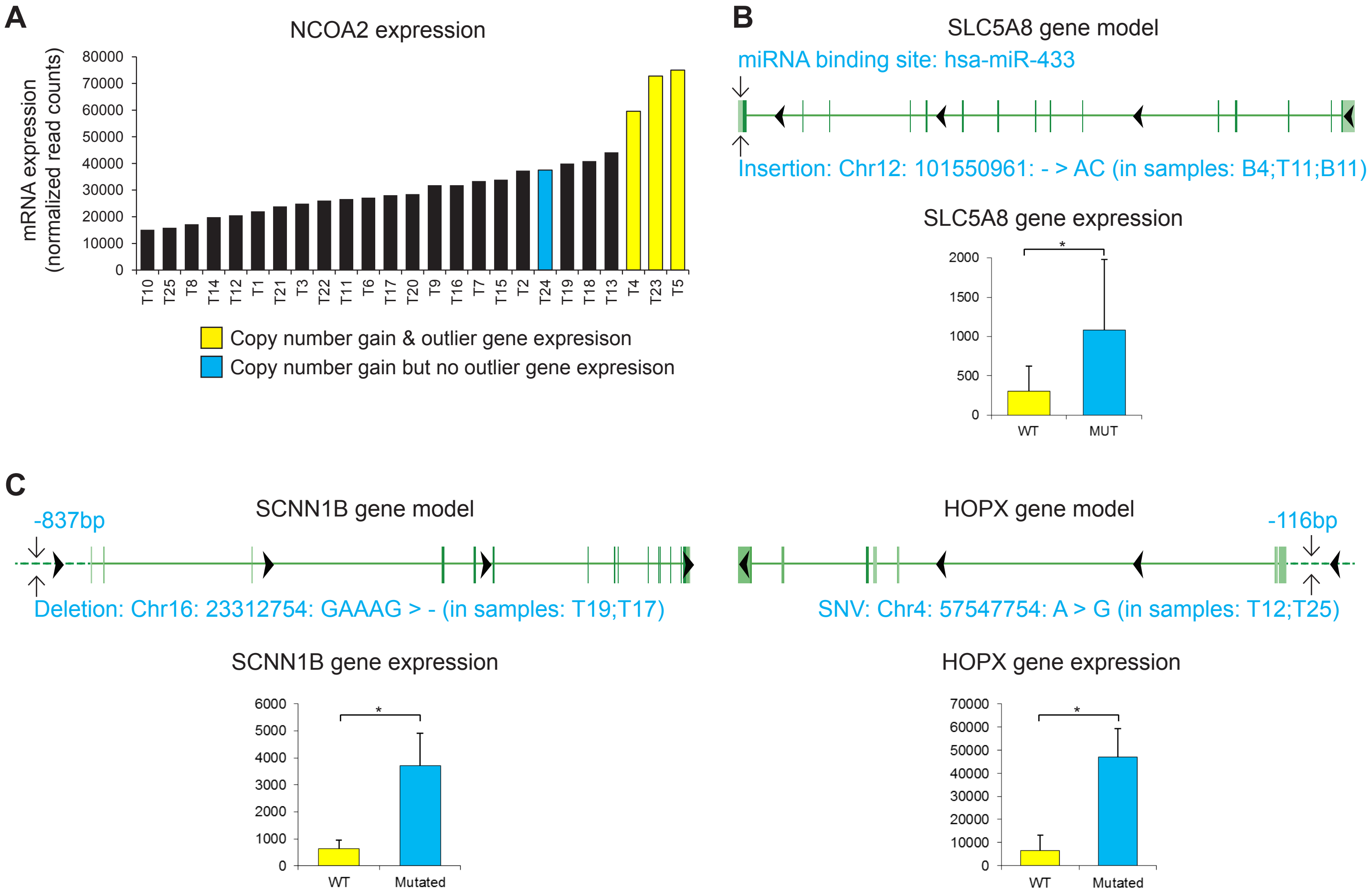

Figure S12: Integration of splice site variants with outlier exon expression. A) Splice site variant within tumor suppressor ERCC2 which results in clear intron retention in tumor T2. Note that in the sashimi plot showing RNA-seq read pile-ups the curved lines represents junction reads. B) A further example of a splice site variant resulting in differential exon expression. In MED16, this particular SNV creates a splice donor site. C) Two examples of SNVs within splice sites that create novel exon-exon junctions. Note that a cancer-relationship is not suggested with the variants in B) and C).

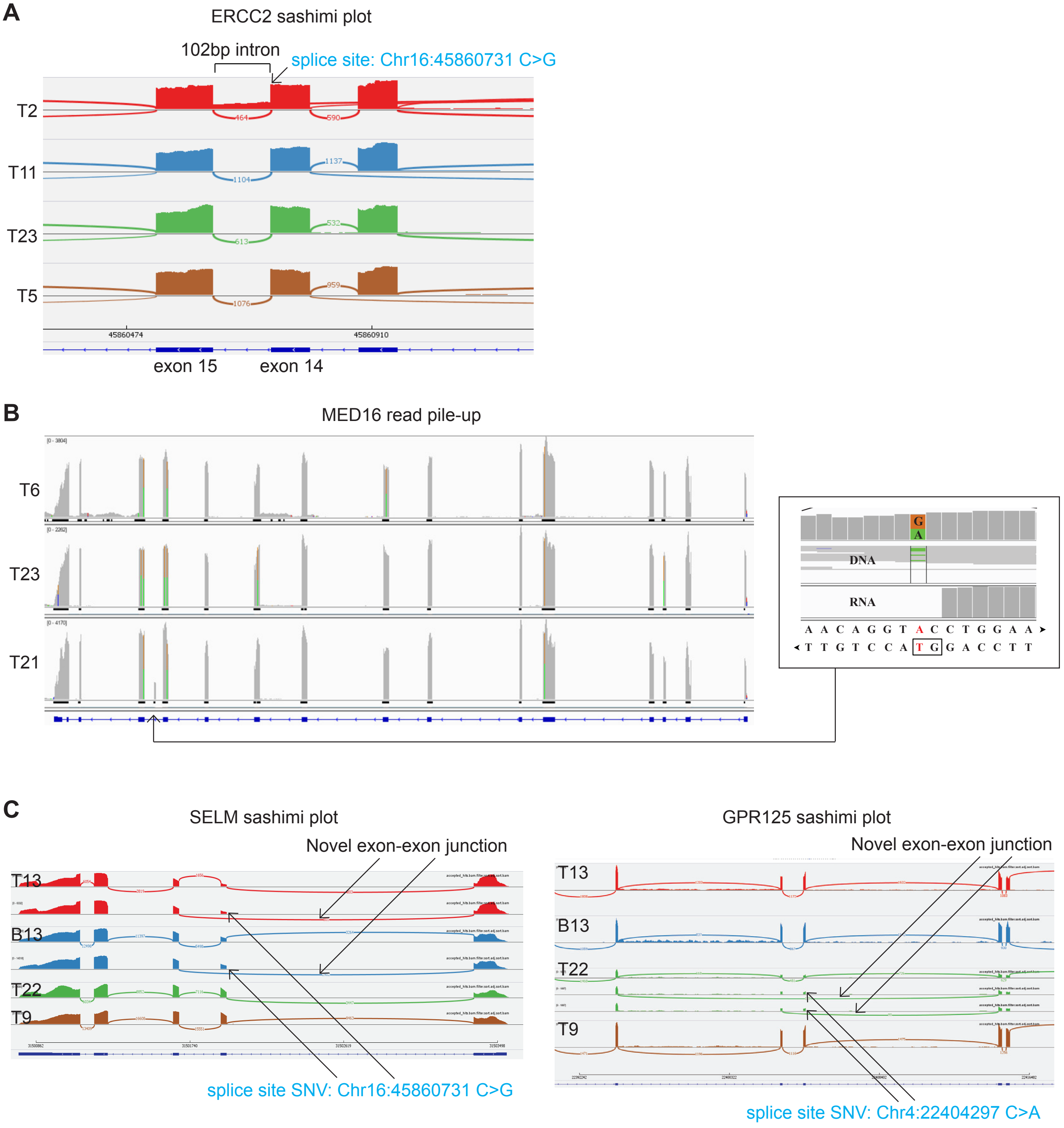

Supplement: Additional file 2: — Figures S1-S10. [file 13059_2014_426_MOESM2_ESM.pdf]
