# Supplementary material for: Heterogeneity in the inter-tumor transcriptome of high risk prostate cancer
Source: Genome Biol. 2014 Aug 26;15(8):426. doi: 10.1186/s13059-014-0426-y (PMC4169643; doi:10.1186/s13059-014-0426-y)
Supplement: Additional file 3: — Text S1. [file 13059_2014_426_MOESM3_ESM.doc]

# Text S1: Supplemental results and discussion

## Global transcript profiling and a signature of therapy response in neo-adjuvant treated tumors

We used DEseq, an R package designed for differential gene expression analysis of RNA-seq data, to quantify transcript expression in our cohort. Global transcript clustering and principal component analyses demonstrated several immediate outliers (Additional File 2: Figure S1A), including T8 and T25 which were likely driven by RNA-Seq related artefacts. T8 was very distant, but inspection of uniquely expressed genes revealed that this effect was likely due to some background DNA sequence contamination within the RNA-seq dataset (note that this observation was controlled for during outlier analyses). T25 was the only sample sequenced on the Illumina GAII platform (compared to HiSeq), highlighting an apparent platform bias. After excluding these samples (including T16, mentioned below) from global comparisons the split between NHT (neo-adjuvant treated) and hormone-naïve tumors became more apparent. We identified 91 transcripts with differential expression between these two groups (DESeq comparison; multiple test corrected *P* value <0.1), 54 transcripts upregulated and 37 downregulated (Additional File 1: Table S4). As expected, most downregulated genes were associated with the AR pathway, and accordingly Ingenuity Pathway Analysis (IPA) predicted the top upstream regulator to be metribolone (activation z-score -3.893; *P* = 2.93×10^-19^). Both DHT and AR were also predicted to be ‘inactivated’, while CCND1 was predicted to be ‘activated’ (activation z-score 3.120; *P* = 1.24×10^-10^). The top function predicted in IPA was prostate cancer (*P* = 2.41×10^-6^), but many functions associated with steroidogenesis (downregulation), lipid metabolism (downregulation), and apoptosis (increased) also scored highly, consistent with the majority of NHT tumors responding to treatment. Other notable upregulated genes included CLU and HSPB1 (Hsp27), which although did not reach the threshold of multiple test corrected significance, are highly functionally and therapeutically relevant .

Although deep transcriptome sequencing provides highly accurate gene expression profiles, analyses are complicated by heterogeneity of input tissue: potentially multi-clonal tumor tissue, benign glands, fibroblast, and lymphocyte populations. These cell types can influence the differential gene expression between samples but often have a reduced correlation with tumor biology or clinical outcome. Given that upregulated genes in NHT tumors could be interpreted as indicators of struggling or dying tumor cells we explored their expression in the MSKCC gene expression (microarray) dataset of 131 primary prostate tumors . In particular we focused on genes associated with apoptosis or growth suppression, for example, CCDC8 (required for p53-mediated apoptosis), CH25H (represses cholesterol synthesis), and DUSP2 (dephosphorylates ERK1 and ERK2). At least one of these genes was upregulated in 15/131 cases, and these cases had longer disease-free survival compared to the rest of the cohort (Logrank *P* = 0.036) (Additional File 2: Figure S1B). Therefore, although we cannot rule out the possibility that the upregulation of some genes after NHT actually reflect a change in (for example) tumoral lymphocyte populations, it appears that the change reflects a biologically-relevant effect. Note that the fact that we were sequencing bulky high-grade disease, rather than smaller low-grade foci, mitigates some concerns surrounding shrinking of tumor foci in response to treatment, as does the unambiguous detection of genome rearrangements and copy number changes in NHT tumors.

We did not observe significant changes in the expression levels of typical fibroblast genes (for example, desmin or vimentin) across the cohort; indeed, no outliers were called for either of these genes. However, one tumor (T3) showed highly significant enrichment of outlier genes in canonical pathways associated with smooth muscle cells (for example, Calcium Signaling, shown in Figure 2). Examples of massive upregulation of ACTA1 (actin) and MYL1 (myosin), representative genes within this pathway are presented in Additional File 2: Figure S4; as are H&E stains from T3 which show clear infiltration of smooth muscle cells. Nevertheless, we still detected robust fusion genes and copy number events in this tumor; evidence that tumor cells still make up the majority of content. Note that one benign sample (B7) also showed enrichment within the Calcium Signaling pathway, albeit to a lower extent.

## Evidence of neuroendocrine transdifferentiation in NHT tumor T20

Global transcript profiling indicated two major outliers which were driven by real biological differences: T16 and T20. The tumor sample T16 was sequenced in a previous study (and re-sequenced here), and was found to have a distinct and unusual neuroendocrine prostate cancer (NEPC) component . As outlined in the main text and Fig. 1D and H, tumor T20 also had a significant NEPC phenotype, which we hypothesized to be driven by exposure to 8 months of neo-adjuvant hormone therapy. Interestingly, even AR positive foci within T20 were highly diffuse with CHGA positive cells, potentially suggesting either ongoing transdifferentiation or the interface of two distinct tumor foci (Additional File 2: Figure S2A). Fusion transcript analysis and PCR validation demonstrated the presence of two different ETS rearrangements in this tumor sample, although junction read counts suggested that the ERG rearrangement was expressed six times higher than the ETV1 rearrangement. FISH showed widespread ERG rearrangement positivity, even in the small cell component of the tumor (Additional File 2: Figure S2C). FISH was unable to demonstrate ETV1 rearrangement positivity suggesting that ETV1-positive cells represent a very minor population. This tumor had an extra copy of MYCN, which is more common in tumors which later become NEPC , but the RB1 genomic loci appeared to be intact: a surprising finding given the very frequent loss of RB1 in NEPC .

## Recurrent mutation to TP53 and FOXA1

Low DNA sequence coverage and absence of matched benign tissue for most patients precluded discovery of novel gene somatic mutations in our cohort. However, the recent series of exome and genome sequencing studies have characterized the coding gene mutational landscape of localized and metastatic prostate cancer . To detect potential driver mutations we considered only non-synonymous, non-dbSNP variants that were detectable simultaneously in DNA and RNA sequence data from the same sample (that is, expressed), and fell within known ‘cancer genes’ from either the Cosmic Cancer Gene Consensus or ‘Mut-Driver Gene’ database . In total there were 49 mutations in 38 cancer genes detected in this manner (Additional File 2: Figure S10; Additional File 1: Table S11), but it is important to note that we cannot resolve somatic from germline changes. Allelic frequency for each mutation is indicated in Additional File 1: Table S11, and was greater than 30% for 43/49 mutations in cancer genes. Significantly recurrent mutations in localized prostate tumors are known to be rare (only SPOP mutations have an incidence >10%), but two tumors in our cohort expressed mutations in FOXA1 (including a nonsense mutation) and three expressed TP53 mutations (meaning TP53 was directly disrupted in over half the cohort (including copy number loss, loss-of-function through gene fusion and ubiquitin ligase upregulation); see Additional File 2: Figure S7). There were no SPOP mutations in our cohort, presumably since we had few ETS negative tumors.

## Integration of genomic variants with outlier gene expression

Paired DNA and RNA sequence data offer an opportunity to identify potential association between genomic changes and outlier gene expression. We performed a series of analyses outlined below to investigate the relative correlation of: (1) copy number variants (CNVs); (2) alterations to microRNA binding sites; (3) changes within promoter regions; and (4) splice site variants; with the set of outlier genes described in the main text and listed in Additional File 1: Table S6. This exploratory analysis is limited by its purely computational nature, and the fact that the majority of changes are likely to be germline. However, the data still provide further insight into transcriptomic complexity.

### Association between outlier gene expression and copy number aberration

We investigated the overlap between CNVs and outlier genes. To call robust and high-confidence CNVs we took the intersection of predictions from aCGH and DNA-seq data. aCGH copy number changes were called using Biodiscovery Nexus CGH software v7, while the tool CNVnator was used for DNA-seq data. In total 479 outlier genes showed an association with CNVs (Fisher’s exact test *P* value <0.05) (Additional File 4: Table S14). Although this meant that in most tumors <1% of outlier genes showed association with CNVs, in tumor T4 almost 10% of outliers were significantly overlapped with CNVs (T4 is the tumor with the tandem duplication genotype). In the cohort as a whole, the gene with the most significant overlap between outliers and CNVs was NCOA2,which was called as an outlier in three tumors, all of which had copy number gain at the NCOA2 loci on chromosome 8 (Additional File 2: Figure S11). NCOA2 is a known AR coactivator, and our data are highly consistent with Taylor *et al.*, who reported that NCOA2 was amplified in primary disease and represents a prostate cancer oncogene .

### Association between outlier gene expression and SNVs within microRNA binding sites

We investigated whether changes to microRNA (miRNA) binding sites were associated with any outlier genes in the cohort. We downloaded miRNA binding sites from the miRanda predictions (http://www.microrna.org/). Next we extracted single nucleotide variants (SNVs) and insertion/deletions (InDels) that fell within these miRNA sites. We retained only those sites which showed a concordant geneotype between DNA and RNA sequence data and had a recurrence frequency less than 50%. Interestingly, there appeared to be a significant association (Chi-square test *P* value: 3.78E-03), between alterations to miRNA binding sites and outlier genes; an association that was not significant when we instead used exonic SNVs/InDels as a negative control (Chi-square test *P* value: 0.1317). At the individual gene level there were 48 SNV/InDels in miRNA binding sites that were significantly overlapped with outlier genes (Fisher’s exact test *P* value <0.05) (Additional File 4: Table S15). We provide an annotated example in Additional File 2: Figure S11. Note that the majority of variants are polymorphic in nature, suggestive of a germline origin.

### Association between outlier gene expression and SNVs within promoter regions

It is plausible that SNVs/InDels affect gene expression through changes to upstream regulatory elements. We extracted SNVs/InDels (with a recurrence frequency less than 50%) up to 1 kb upstream of the transcriptional start site (including the 5’ UTR) and overlapped these changes with outlier gene expression results. Again, there appeared to be a significant association between alterations to ‘promoter’ regions and outlier expression (Chi-square test *P* value: 1.7E-04), which was absent from the exonic SNVs/InDels negative control (Chi-square test *P* value: 0.1317). In total we detected 169 SNV/InDels which significantly overlapped with outlier gene expression (Fisher’s exact test *P* value <0.05) (Additional File 4: Table S16). We provide annotated examples in Additional File 2: Figure S11.

### Association between SNVs and differential splice isoforms

Changes to splice sites (for example, 2 bp flanking region of exons) can result in differential splicing. Our paired DNA and RNA-seq data provided an opportunity to explore the association of outlier splicing with splice site SNVs/InDels. First we identified outlier splice exons and junctions. We calculated the read counts of adjacent exons and involved junctions and normalized against the total read counts of the corresponding genes. Next we applied the same recurrent outlier analysis method as was performed for whole genes in the main manuscript (GESD, exon coverage > 30X, junction coverage > 15X, and gene coverage > 15X). Only those exons or junctions involved in protein domains or structural features (using Ensembl annotation) were retained for further analysis. Interestingly, we observed a significant overlap between outlier exons/junctions and splice site SNVs/InDels (that were present in <50% of the cohort) (Chi-square test *P* value: 3.66E-69). As a control we performed the same analysis using SNVs/InDels within 2 bp of exonic boundaries. Using this ‘decoy set’ we did not observe a significant association (Chi-square test *P* value: 0.084). In total there were 49 SNVs/InDels in 46 genes that were associated with a corresponding exon/junction outlier (Fisher’s exact test *P* value <0.05) (Additional File 4: Table S17). Two examples are provided in (Additional File 2: Figure S12), including a splice site variant within ERCC2 (tumor suppressor involved in DNA repair) which is likely to result in a defective gene product. We also observed several SNVs/InDels which were associated with novel exon-exon junctions (Additional File 4: Table S18; Additional File 2: Figure S12).

## References

1. Matsumoto H, Yamamoto Y, Shiota M, Kuruma H, Beraldi E, Matsuyama H, Zoubeidi A, Gleave M: **Cotargeting androgen receptor and clusterin delays castrate-resistant prostate cancer progression by inhibiting adaptive stress response and AR stability.** *Cancer Res* 2013, **73:**5206-5217.

2. Zoubeidi A, Zardan A, Wiedmann RM, Locke J, Beraldi E, Fazli L, Gleave ME: **Hsp27 promotes insulin-like growth factor-I survival signaling in prostate cancer via p90Rsk-dependent phosphorylation and inactivation of BAD.** *Cancer Res* 2010, **70:**2307-2317.

3. Loriot Y, Zoubeidi A, Gleave ME: **Targeted therapies in metastatic castration-resistant prostate cancer: beyond the androgen receptor.** *Urol Clin North Am* 2012, **39:**517-531.

4. Taylor BS, Schultz N, Hieronymus H, Gopalan A, Xiao Y, Carver BS, Arora VK, Kaushik P, Cerami E, Reva B, Antipin Y, Mitsiades N, Landers T, Dolgalev I, Major JE, Wilson M, Socci ND, Lash AE, Heguy A, Eastham JA, Scher HI, Reuter VE, Scardino PT, Sander C, Sawyers CL, Gerald WL: **Integrative genomic profiling of human prostate cancer.** *Cancer Cell* 2010, **18:**11-22.

5. Wu C, Wyatt AW, Lapuk AV, McPherson A, McConeghy BJ, Bell RH, Anderson S, Haegert A, Brahmbhatt S, Shukin R, Mo F, Li E, Fazli L, Hurtado-Coll A, Jones EC, Butterfield YS, Hach F, Hormozdiari F, Hajirasouliha I, Boutros PC, Bristow RG, Jones SJ, Hirst M, Marra MA, Maher CA, Chinnaiyan AM, Sahinalp SC, Gleave ME, Volik SV, Collins CC: **Integrated genome and transcriptome sequencing identifies a novel form of hybrid and aggressive prostate cancer.** *J Pathol* 2012, **227:**53-61.

6. Beltran H, Rickman DS, Park K, Chae SS, Sboner A, MacDonald TY, Wang Y, Sheikh KL, Terry S, Tagawa ST, Dhir R, Nelson JB, de la Taille A, Allory Y, Gerstein MB, Perner S, Pienta KJ, Chinnaiyan AM, Wang Y, Collins CC, Gleave ME, Demichelis F, Nanus DM, Rubin MA: **Molecular characterization of neuroendocrine prostate cancer and identification of new drug targets.** *Cancer Discovery* 2011, **1:**487-495.

7. Mosquera JM, Beltran H, Park K, MacDonald TY, Robinson BD, Tagawa ST, Perner S, Bismar TA, Erbersdobler A, Dhir R, Nelson JB, Nanus DM, Rubin MA: **Concurrent AURKA and MYCN gene amplifications are harbingers of lethal treatment-related neuroendocrine prostate cancer.** *Neoplasia* 2013, **15:**1-10.

8. Tan HL, Sood A, Rahimi HA, Wang W, Gupta N, Hicks J, Mosier S, Gocke CD, Epstein JI, Netto GJ, Liu W, Isaacs WB, De Marzo AM, Lotan TL: **Rb loss is characteristic of prostatic small cell neuroendocrine carcinoma.** *Clin Cancer Res* 2014, **20:**890-903.

9. Barbieri CE, Baca SC, Lawrence MS, Demichelis F, Blattner M, Theurillat JP, White TA, Stojanov P, Van Allen E, Stransky N, Nickerson E, Chae SS, Boysen G, Auclair D, Onofrio RC, Park K, Kitabayashi N, MacDonald TY, Sheikh K, Vuong T, Guiducci C, Cibulskis K, Sivachenko A, Carter SL, Saksena G, Voet D, Hussain WM, Ramos AH, Winckler W, Redman MC, *et al*: **Exome sequencing identifies recurrent SPOP, FOXA1 and MED12 mutations in prostate cancer.** *Nat Genet* 2012, **44:**685-689.

10. Grasso CS, Wu YM, Robinson DR, Cao X, Dhanasekaran SM, Khan AP, Quist MJ, Jing X, Lonigro RJ, Brenner JC, Asangani IA, Ateeq B, Chun SY, Siddiqui J, Sam L, Anstett M, Mehra R, Prensner JR, Palanisamy N, Ryslik GA, Vandin F, Raphael BJ, Kunju LP, Rhodes DR, Pienta KJ, Chinnaiyan AM, Tomlins SA: **The mutational landscape of lethal castration-resistant prostate cancer.** *Nature* 2012, **487:**239-243.

11. Baca SC, Prandi D, Lawrence MS, Mosquera JM, Romanel A, Drier Y, Park K, Kitabayashi N, MacDonald TY, Ghandi M, Van Allen E, Kryukov GV, Sboner A, Theurillat JP, Soong TD, Nickerson E, Auclair D, Tewari A, Beltran H, Onofrio RC, Boysen G, Guiducci C, Barbieri CE, Cibulskis K, Sivachenko A, Carter SL, Saksena G, Voet D, Ramos AH, Winckler W *et al*: **Punctuated evolution of prostate cancer genomes.** *Cell* 2013, **153:**666-677.

12. Futreal PA, Coin L, Marshall M, Down T, Hubbard T, Wooster R, Rahman N, Stratton MR: **A census of human cancer genes.** *Nat Rev Cancer* 2004, **4:**177-183.

13. Vogelstein B, Papadopoulos N, Velculescu VE, Zhou S, Diaz LA, Jr, Kinzler KW: **Cancer genome landscapes.** *Science* 2013, **339:**1546-1558.

14. Abyzov A, Urban AE, Snyder M, Gerstein M: **CNVnator: an approach to discover, genotype, and characterize typical and atypical CNVs from family and population genome sequencing.** *Genome Res* 2011, **21:**974-984.
